# Habituation of the stress response multiplex to repeated cold pressor exposure

**DOI:** 10.3389/fphys.2022.752900

**Published:** 2023-01-10

**Authors:** Tom Bullock, Mary H. MacLean, Tyler Santander, Alexander P. Boone, Viktoriya Babenko, Neil M. Dundon, Alexander Stuber, Liann Jimmons, Jamie Raymer, Gold N. Okafor, Michael B. Miller, Barry Giesbrecht, Scott T. Grafton

**Affiliations:** ^1^ Department of Psychological and Brain Sciences, University of California, Santa Barbara, Santa Barbara, CA, United States; ^2^ Institute for Collaborative Biotechnologies, University of California, Santa Barbara, Santa Barbara, CA, United States; ^3^ Department of Child and Adolescent Psychiatry, Psychotherapy and Psychosomatics, University of Freiburg, Freiburg im Breisgau, Germany

**Keywords:** stress, cold pressor, habituation, adaptation, cardiac physiology, EEG, pupillometry, repeated exposure

## Abstract

Humans show remarkable habituation to aversive events as reflected by changes of both subjective report and objective measures of stress. Although much experimental human research focuses on the effects of stress, relatively little is known about the cascade of physiological and neural responses that contribute to stress habituation. The cold pressor test (CPT) is a common method for inducing acute stress in human participants in the laboratory; however, there are gaps in our understanding of the global state changes resulting from this stress-induction technique and how these responses change over multiple exposures. Here, we measure the stress response to repeated CPT exposures using an extensive suite of physiologic measures and state-of-the-art analysis techniques. In two separate sessions on different days, participants underwent five 90 s CPT exposures of both feet and five warm water control exposures, while electrocardiography (ECG), impedance cardiography, continuous blood pressure, pupillometry, scalp electroencephalography (EEG), salivary cortisol and self-reported pain assessments were recorded. A diverse array of adaptive responses are reported that vary in their temporal dynamics within each exposure as well as habituation across repeated exposures. During cold-water exposure there was a cascade of changes across several cardiovascular measures (elevated heart rate (HR), cardiac output (CO) and Mean Arterial Pressure (MAP) and reduced left ventricular ejection time (LVET), stroke volume (SV) and high-frequency heart rate variability (HF)). Increased pupil dilation was observed, as was increased power in low-frequency bands (delta and theta) across frontal EEG electrode sites. Several cardiovascular measures also habituated over repeated cold-water exposures (HR, MAP, CO, SV, LVET) as did pupil dilation and alpha frequency activity across the scalp. Anticipation of cold water induced stress effects in the time-period immediately prior to exposure, indexed by increased pupil size and cortical disinhibition in the alpha and beta frequency bands across central scalp sites. These results provide comprehensive insight into the evolution of a diverse array of stress responses to an acute noxious stressor, and how these responses adaptively contribute to stress habituation.

## 1 Introduction

Repeated exposures to aversive stimuli may result in attenuation of physiological responses associated with pain or stress in the human. However, relatively little is known about the multiplex of physiological and neural responses that adaptively contribute to stress habituation. Much previous work measures select responses (e.g., cardiovascular, endocrine) to singular exposures of stress in human participants, and as a result there are gaps in our understanding of both the global state changes that result from stress induction and also how these responses habituate over repeated exposures. We take advantage of a dataset recorded as part of a larger study into the effects of stress on human brain and behavioral responses to investigate habituation to a common stress induction technique, the cold pressor test (CPT; Hines and Brown, 1932). Here we report results from a diverse array of physiological and neural measures across multiple CPT exposures to gain new insight into habituation to an acute stressor.

The CPT induces stress in humans by immersing a limb or limbs in cold water, typically for between 90 s and 3 min (Hines and Brown, 1932; Al’Absi et al., 2002; Suter et al., 2007; Frings et al., 2013; Larra et al., 2015). The pattern of physiological responses evoked by the CPT can be best understood as adaptations that prepare the human to respond a threat (i.e., the pain/discomfort of cold-water exposure) by either fight or flight. These adaptations are evoked by many different forms of environmental and mental stressors and are collectively referred to as the defense reaction, broadly comprising an increase in cardiovascular output, vasodilation of the skeletal muscles and vasoconstriction in the skin, kidneys and guts (Hilton, 1982; Zbrozyna and Westwood, 1993). Specifically, exposure to the CPT evokes a range of autonomic effects, including elevated HR, systolic and diastolic blood pressure, CO, TPR and decreased LVET (e.g., Lovallo 1975; Larra et al., 2015; Bachmann et al., 2018). The CPT often, but not always, evokes vasodilation in the calf and forearm muscles during immersion of a single foot in cold water, and vasoconstriction in the hand, with muscle vasodilation habituating more readily upon repeated CPT exposure (Zbrożyna and Westwood, 1988, 1990; Edwards et al., 1999). Increased pupil size (Tassorelli et al., 1995; Tassorelli et al., 1998), skin conductance responses (Engel, 1959; Buchanan et al., 2006) and elevated levels of epinephrine and norepinephrine have also been documented in response to the CPT (Winer and Carter, 1977; Woolf et al., 1983; Hassellund et al., 2010). Furthermore, hypothalamic-pituitary-adrenal (HPA) axis activation, assayed with glucocorticoid activity (cortisol), is sometimes evoked by the standard CPT (Errico et al., 1993; Pascualy et al., 2000; Al’Absi et al., 2002) but not always (McRae et al., 2006; Duncko et al., 2007; Skoluda et al., 2015).

There is considerable evidence from the non-human animal literature that the defense response is integrated in specific and well-localized areas of the ventral hypothalamus, basal amygdala, midbrain and medulla (Hilton, 1982) and modulated by specific regions of prefrontal cortex (Al Maskati and Zbrozyna, 1989; Zbrozyna and Westwood, 1993). In the human, the amygdala and bed nucleus of the stria terminalis (BNST) are implicated in the defense response, with evidence implicating BNST in threat anticipation and the amygdala in responding to acute danger (Straube et al., 2007; Alvarez et al., 2011; Klumpers et al., 2017). Prefrontal cortex likely plays an important role in habituation of the defense response to repeated stressor exposure. Evidence comes from experiments with rats where lesions in prefrontal cortex impaired habituation of cardiovascular responses whereas stimulation of the same areas enhanced habituation (Glaser and Griffin, 1962) and experiments where stimulation of a specific region of prefrontal cortex inhibits tachycardia, vasodilation in the muscles and vasoconstriction in the kidneys (Al Maskati and Zbrozyna, 1989; Zbrozyna and Westwood, 1993). In the human, changes in brain oscillatory activity as a function of cold-water exposure have been reported (Chen et al., 1989; Backonja et al., 1991; Chen and Rappelsberger, 1994; Ferracuti et al., 1994; Chang et al., 2002; Zhang et al., 2018), but whether or not oscillatory activity demonstrates adaptive changes or habituation to multiple stressor exposures is unknown.

The main goal of the present study was to address two important gaps in the current CPT literature. First, CPT effects on both autonomic nervous system (ANS) and central nervous system (CNS) function are often reported in cross-sectional studies (e.g., Lovallo 1975; Backonja et al., 1991; Ferracuti et al., 1994; Tassorelli et al., 1995; Chang, Arendt-Nielsen, and Chen 2002) with few simultaneous measures (e.g., Zhang et al., 2018). Accordingly, the simultaneous time course of different measures (e.g., autonomic and neural) within-individuals remains undocumented. Second, the effect of repeated CPT exposures on a diverse array of simultaneously recorded autonomic and neural responses is unclear. While several previous studies have implemented multiple CPT exposures within a single participant, responses were limited to measures of brain oxygenation (Barati et al., 2013) and basic cardiovascular measures paired with self-report (Glaser and Whittow, 1957; Glaser et al., 1959; Stancák et al., 1996) and muscle blood flow (Zbrożyna and Krebbel, 1985; Zbrożyna and Westwood, 1988, 1990). There are also substantial differences between the within-session testing protocols used by different investigations, with CPT exposure durations varying from 45 s (Barati et al., 2013) to 4 min (Stancák et al., 1996), repetitions between three exposures (Barati et al., 2013) and ten exposures (Glaser and Whittow, 1957) and inter-exposure recovery periods from 60 s (Glaser and Whittow, 1957) to 10 minutes (Zbrożyna and Krebbel, 1985). These previous investigations undoubtedly provide valuable information on CPT habituation, but the limited simultaneous measures used within each study and variation between CPT protocols means that it is not possible to gain a comprehensive overview of how adaptation processes unfold both within and between repeated CPT exposures. The present study contributes to a richer understanding of CPT habituation by examining multiple physiological and neural measures simultaneously during five repeated CPT exposures to gain insight into the evolving multiplex of acute stress habituation.

## 2 Methods

### 2.1 Participant recruitment and preliminary assessment

Healthy adults aged 18 to 35 were recruited as part of the Biomarkers of Stress States (BOSS) study run at the University of California, Santa Barbara with the goal of investigating how different types of acute stress (noxious stimulation, social stress, mental fatigue and physical fatigue) impact human physiology, brain and behavior. All participants were initially pre-screened *via* phone interview to determine their eligibility to participate. Participants were considered ineligible if any of the following criteria applied: heart condition or joint issues, recent surgeries that would inhibit movement, BMI>30, currently taking blood pressure medication or any psychostimulants or antidepressants. Participants also agreed to provide a blood sample and multiple saliva samples throughout the study. Participants were instructed to eat and drink in accordance with their typical daily routine and they were not specifically instructed to abstain from caffeinated beverages. They were instructed to abstain from strenuous exercise in the 48 h prior to each testing session. Informed consent was provided at the beginning of each session, and all procedures were approved by Western IRB and the U.S. Army Human Research Protection Office, and conformed to UC Santa Barbara Human Subjects Committee policies.

Participants were then invited to attend the lab to complete two preliminary testing sessions on two separate days. In session one, all participants underwent a trial 90 s CPT exposure (described in Section 3.3) to determine whether they were able to withstand the discomfort associated with the CPT, and participants who were unable to last the full 90 s were not allowed to participate in subsequent sessions (*n* = 1). In session two, participants completed a graded VO_2_max test on a stationary exercise bike to assess their aerobic fitness capacity (Vyntus CPX Metabolic Cart and Viasprint 150P stationary bike, Vyaire Medical, Yorba Linda, CA, United States). VO_2_max data are reported here, but were primarily collected as part of the larger BOSS study for another purpose.

A subset of *n* = 42 participants were then randomly allocated to the noxious stimulation (CPT) condition. Participant age, sex and physiological data are reported in Table 1. Participants were all members of the UC Santa Barbara campus community and were either current students (*n* = 34), had recently graduated and were in full-time employment (*n* = 5) or were unemployed (*n* = 4). The ethnic background of the sample was as follows: white/Caucasian (*n* = 16), Asian (*n* = 9), Hispanic/Latino/Spanish Origin (*n* = 6), black/African-American (*n* = 3), Middle-Eastern/North-African (*n* = 2), White/Hispanic (*n* = 3), White/Asian (*n* = 1), Hispanic/Asian (*n* = 1), other (*n* = 2). The total household annual income reported by participants was as follows: <$20,000 (*n* = 5), $20,000 to $34,999 (*n* = 4), $35,000 to $49,999 (*n* = 6), $50,000 to $74,999 (*n* = 5), $75,000 to $99,999 (*n* = 7), > $100000 (*n* = 10). Six participants declined to report annual household income. Data collected from participants assigned to other stress conditions as part of the larger BOSS protocol are not reported in this manuscript. All participants received compensation of $20 per hour for the time they spent in the lab, with an additional $25 bonus for completing the entire BOSS protocol.

**TABLE 1:** Participant demographic and baseline cardiovascular data, computed from the mean of the final 15 s of the baseline period in first trial of the control condition. Mean (SD).

	*n*	Age	BMI	VO2_max_	HR	HF	PEP	LVET	CO	SV	MAP	SBP	DBP	TPR
Males	22	20 (1.80)	23.78 (5.19)	41.71 (8.79)	68.69 (15.61)	6.28 (1.12)	79.07 (9.77)	279.49 (29.16)	2.39 (0.68)	36.21 (10.13)	83.18 (13.00)	108.56 (15.54)	67.42 (12.03)	3118 (1434)
Females	20	20.7 (2.64)	22.95 (2.42)<	37.97 (7.18)	63.16 (11.87)	6.45 (1.51)	87.02 (12.69)	296.43 (26.80)	2.39 (0.64)	39.09 (13.09)	77.08 (11.79)	97.33 (20.42)	65.86 (9.11)	2757 (814)
All	42	20.35 (2.24)	23.37 (4.08)	39.84 (8.19)	66.01 (13.99)	6.35 (1.31)	82.93 (11.81)	287.67 (28.92)	2.39 (0.65)	37.61 (11.56)	80.12 (12.58)	102.95 (18.73)	66.64 (10.52)	2938 (1161)

Given that the main goal of the larger BOSS study was to investigate stress effects on cognitive performance and underlying physiology, sample size was estimated based on what is typical in the literature for cognitive tasks and not investigations into CPT effects *per se*. However, the present sample size exceeds the sample size of comparable CPT investigations. For example, two recent investigations validating the “both feet” version of the CPT used in this study sampled 24 participants (Larra et al., 2015) and 28 participants (Bachmann et al., 2018) and investigations into CPT habituation effects test between 5 and 28 participants (Glaser and Whittow, 1957; Glaser et al., 1959; Zbrożyna and Krebbel, 1985; Zbrożyna and Westwood, 1988, 1990; Stancák et al., 1996; Barati et al., 2013).

### 2.2 Instrumentation and general procedure

In sessions three, four and five, participants attended the lab and completed a sequence of cognitive tasks while physiological and neural measures were recorded. Each session was completed on a separate day. Session three was a baseline recording (i.e., no stress manipulation) whereas session four involved a stress manipulation and session five involved an active control manipulation. Sessions four and five were completed in a counterbalanced order (order was randomly assigned based on participant number). In this paper, data are reported from sessions four and five only.

Each participant started both sessions at either 8a.m. or 1p.m. On both days, participants were informed at the start of the session which condition they would be exposed to (i.e. treatment or control) and that there would be five cold water exposure (CPT) or five warm water exposure (warm pressor test; WPT) trials in total. Participants were then prepared for the physiological recording by fitting disposable foam electrodes (EL500, BIOPAC Systems Inc., Goleta, CA, United States) to the neck and torso. Prior to the placement of each electrode, an approximate one-inch area of skin was disinfected and gently exfoliated with an abrasive pad, followed by application of Nu-Prep exfoliation gel (EVPREP, BIOPAC). The area was fanned dry and then a small amount of electrode gel (GEL100, BIOPAC) was placed on each electrode before being placed on participants. For collection of impedance cardiography (ICG), 8 electrodes were placed on the torso and neck, two on each side of the neck and two on each side of the torso (Bernstein, 1986). For ECG, two electrodes were placed on the chest, one under the right collarbone and one under the left rib cage. A total of 10 electrodes were applied.

**FIGURE 1:**
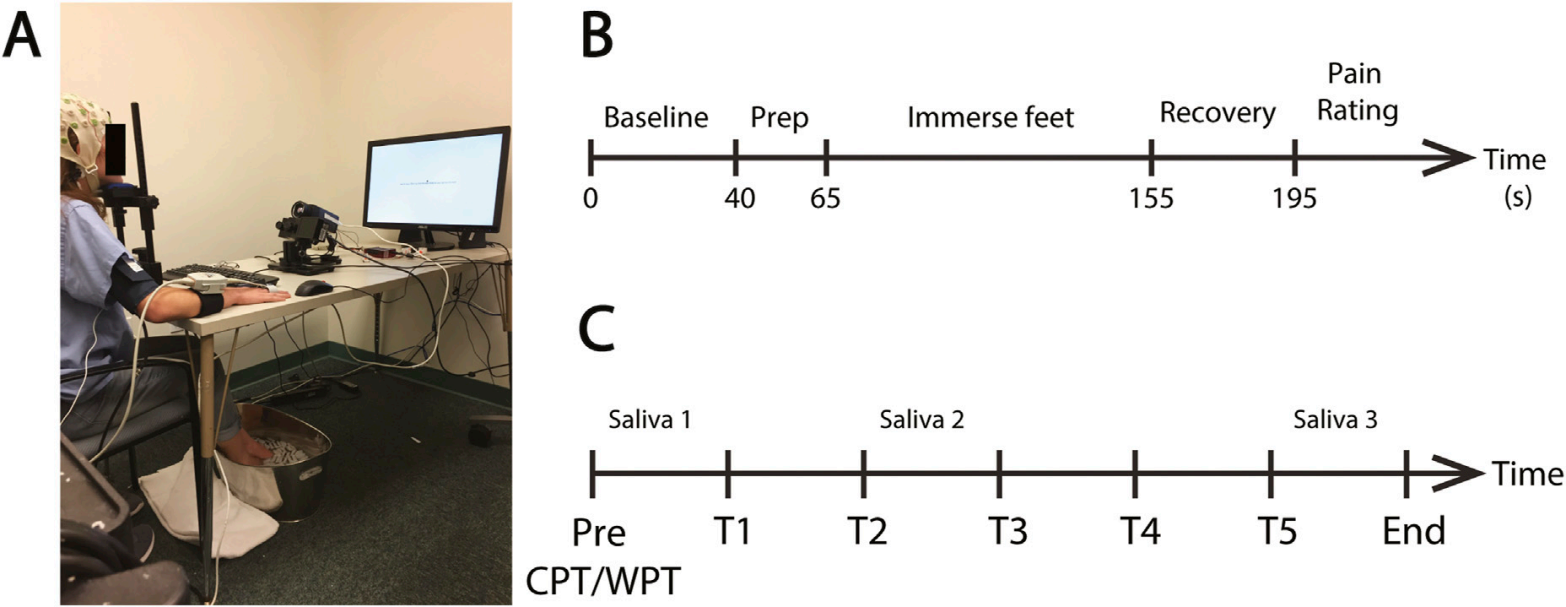
Pressor protocol. **(A)** An example of a fully instrumented participant in the treatment condition. **(B)** The standard protocol for each CPT or WPT trial. Each protocol consisted of a pre-immersion baseline recording (40 s) followed by a preparatory period (25 s) where the participant was required to position their feet on the edge of the water bucket in preparation for immersion. The participant then lowered their feet into the water (90 s) and removed them from the water for the recovery period (40 s). They then provided a pain rating. **(C)** Schematic of the complete experiment protocol. Participants completed five CPT exposures on the Treatment Day or five WPT exposures on the Control Day (T1—T5). The time between the onset of each exposure was ∼25 min. Saliva samples were collected prior to T1 and T3 and immediately after T5.

Participants were then fitted with an EEG cap consisting of 63 scalp electrodes arranged in accordance with the 10–20 system (BrainAmp MR, Brain Products, Berlin, Germany) and positioned at a desk in front of a computer monitor (120 cm viewing distance). A CNAP Monitor 500 (BIOPAC) blood pressure cuff was attached to the right upper arm and a finger cuff to the middle and index fingers for continuous blood pressure (CBP) monitoring. An eye-tracker (Eyelink 1000 Plus, SR Research, Ltd., Mississauga, Ontario, Canada) was positioned on the desk at ∼60 cm distance from the participant’s eyes and an infrared camera (FLIR Systems, Goleta, CA, United States) was positioned at ∼65 cm distance. The infrared camera data were recorded for another purpose (Kumar et al., 2021). The participant rested on a chin support (SR Research, Ltd.) in order to stabilize the head. A fully instrumented participant is shown in Figure 1A.

Participants then underwent five CPT immersions in the treatment session and five WPT sessions in the control session. The CPT/WPT protocol is detailed in Figure 1B and described in the next section. Each individual CPT/WPT immersion within a session is referred to as a “Trial”, using T1-T5 as shorthand to denote Trials 1–5. Session order was counterbalanced between participants. Immediately following each trial, participants engaged in a cognitive task as part of the BOSS protocol (data pertaining to the cognitive tasks were collected for another purpose and are not reported here). Each cognitive task had a different duration, and time to produce each of the saliva samples varied between subjects, so time between CPT/WPT exposures was variable (mean = 24 m 55 s, SD = 310 s). The variability in time between CPT/WPT exposures and the potential for the cognitive tasks to impact the stress response are limiting factors of this dataset, and this is explored in the “Limitations and Future Directions” section of the discussion. The complete experimental session structure is outlined in Figure 1C.

Participants were allowed to drink water during the protocol and they were allowed to take bathroom breaks in between tasks/CPT exposures.

### 2.3 Cold pressor test procedure

A version of the CPT/WPT where participants immersed both feet in water was chosen for two key reasons. First, participants needed to be able to use both hands immediately after the termination of the CPT to make responses to the cognitive tasks, hence immersing the hands was not suitable as numbness in the hands may impair task performance. Second, recent evidence suggests that the stress response may be enhanced when participants are required to immerse both feet in water when compared to single arm (Larra et al., 2015). This version of CPT has been used in several recent studies and is well validated (Bachmann et al., 2018; Zhang et al., 2018).

For each trial, a large metal bucket was positioned just in front of the participant’s feet and a towel was placed under the feet. A digital thermometer was positioned in the bucket. For treatment sessions, the bucket was pre-filled with cold water and ice at least 15 min prior to the first exposure (∼33 °f/0.6°C) whereas for control sessions the bucket was pre-filled with lukewarm water (∼92 °f/33.3°C). The bucket was filled to ∼10 cm deep. The water was stirred between trials and measured between trials to ensure that a consistent temperature was maintained throughout the session. The temperature in the laboratory was thermostatically maintained at ∼ 22°C during the protocols.

A blood pressure cuff was fitted to the right hand/arm and calibrated prior to starting the recording. A fixation cross was then presented on screen. The participant was instructed to fixate on this cross throughout the duration of the experiment and remain as still and relaxed as possible throughout the entire procedure. The protocol was then initiated (Figure 1B). The protocol started with a baseline period (40 s) where the participant was just required to relax and maintain their gaze on the fixation cross. Next, a tone sounded and a written instruction appeared asking the participant to position their feet on the edge of the bucket in preparation for immersion (25 s). A tone then sounded and the participant was instructed to immerse their feet in the bucket and try to relax and stay as still as possible (90 s). At the end of the immersion period, another tone sounded and the participant was instructed to remove both feet from the bucket and place them on a towel for the recovery period (40 s) and try to relax and minimize any further movement. At the end of the recovery period the participant was asked to report the amount of pain they had experienced on a scale from 0 (no pain) to 100 (extremely painful). Upon completion of the procedure the participant was then given the opportunity to dry and wrap their feet in a dry towel for comfort, until the next trial. Importantly, during the baseline, CPT and recovery periods participants were instructed to keep their feet flat on the ground or the bottom of the ice bucket and to remain as still as possible in order to obtain steady and reliable blood pressure, ECG and impedance cardiograph readings.

### 2.4 Measurements

During each trial, EEG was recorded with 63 scalp electrodes at 1000 Hz sampling rate (BrainAmp MR, Brain Products). ICG, ECG and CBP were sampled at 4000 Hz with an MP150 Amplifier and displayed and stored on a laptop running AcqKnowledge Software Version 5.0.2 (BIOPAC Systems, Inc.). Binocular pupillometry was sampled at 500 Hz (Eyelink 1000 Plus, SR Research). Saliva samples were collected at three points throughout each session: one at the beginning of the experiment, one after the second CPT/WPT and one after the final CPT/W*p*T. Samples were collected using the passive drool technique, where the participant was required to salivate through a straw into a vial. Saliva samples were transferred to a freezer (-80°C) at the end of each session.

### 2.5 Data pre-processing

#### 2.5.1 Cardiovascular measures

**FIGURE 2:**
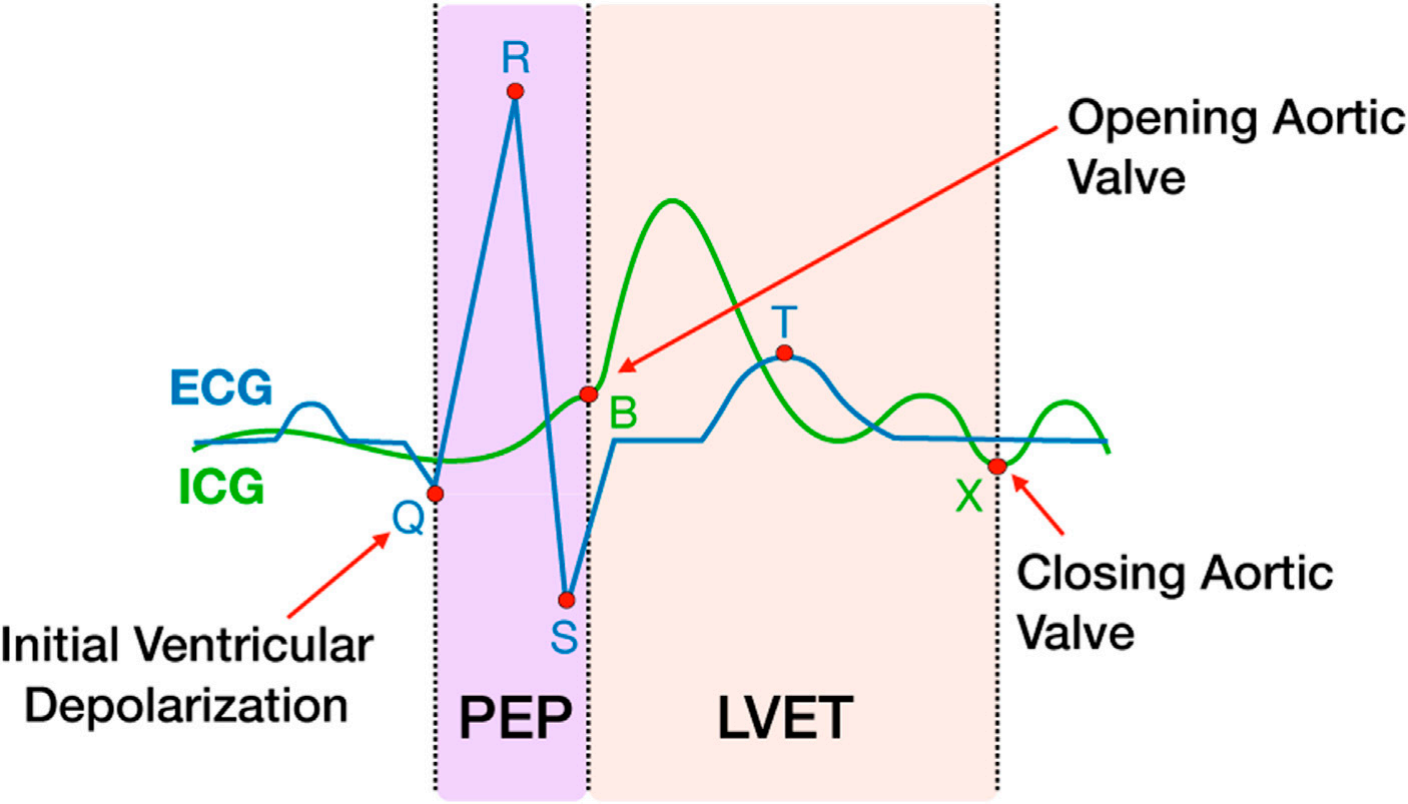
PEP and LVET measures extracted from ICG and ECG waveforms. The ICG waveform (green line) is shown overlapping the ECG waveform (blue line). The ICG B and X points represent the open and closing of the aortic valve, respectively. The Q point of the ECG represents the initial ventricular depolarization. The time interval between Q and B represents PEP, the sum of electromechanical delay and isovolumic contraction of the ventricle. LVET is the time interval between B and X.

Physio data were downsampled to 2000 Hz and pre-processed and analyzed using the Moving Ensemble Analysis Pipeline (MEAP Software Version 1.5.3a, Cieslak et al., 2018). The outputs were moving-ensemble averaged across a 15 s window, allowing for continuous estimations of physiological measures of mean arterial blood pressure (MAP), heart rate (HR), pre-ejection period (PEP), total peripheral resistance (TPR), left ventricular ejection time (LVET), cardiac output (CO) and stroke volume (SV). PEP was calculated using a combination of ICG and ECG, as the time interval between the first ventricular depolarization and the moment blood is ejected from the left ventricle. Shorter PEP time intervals represent increased sympathetic activation (Figure 2). LVET was calculated as the time interval between the opening and closing of the aortic valve. SV represents the volume of blood in mL pumped from the left ventricle per heartbeat. CO represents the amount of blood pumped through the circulatory system per minute. TPR, also known as systemic vascular resistance, is derived from CO and mean arterial pressure and represents the resistance applied to whole body blood flow through the circulatory system. An estimate of respiration was also computed using the ICG signal and modeled out of all the cardiovascular measures as part of the MEAP preprocessing to remove low-frequency fluctuations in the signal caused by respiration and not blood flow (Cieslak et al., 2018). Three expertly trained research assistants scored the data in MEAP. To control for reliability across research assistants scoring the data, the same research assistant analyzed all CPT trials for each participant.

Variability in R-R intervals is commonly separated into low (0.04–0.15 Hz) and high (0.15–0.40 Hz) frequencies, with the former reflecting a complex mixture of sympathetic and parasympathetic tone (Saul et al., 1991) and the latter serving as a proxy of parasympathetic cardiac tone (Barbieri et al., 2005). High frequency heart rate variability (HF) was characterized with point process estimation software developed by Riccardo Barbieri and Luca Citi, executed as MATLAB code (available at http://users.neurostat.mit.edu/barbieri/pphrv). In brief, the code uses inverse Gaussian regression and spectral analysis of the latencies of ECG R points to generate a continuous estimation of HF (Chen and Kuo, 2007) i.e., power spectral density between 0.15 and 0.40 Hz, expressed in ms^2^/Hz. Each pointwise estimate is trained on the 32 s of R points immediately preceding it (note in Figure 4 there are no HF estimates for 80% of the baseline period). The logarithmic transform of the estimated HF time series was extracted as a proxy of parasympathetic input to the heart, with higher values reflecting increased activity in this autonomic branch. For consistency with the ICG measures, parasympathetic estimates were also corrected for respiratory state. This correction was applied to account for known influences of respiratory activity on heart rate and vagal tone (Larsen et al., 2010) computed in the current pipeline from raw ECG R points. Previous work that has used the Barbieri & Cita MATLAB code to generate a continuous estimate of HF has also applied a respiration correction (e.g., Dundon et al., 2020). Respiratory state was defined as the normalized product of the phase and magnitude of respiration activity (estimated from the raw impedance time series) at each R point. Respiration corrected measures of HF were the residuals from a linear model of raw HF against respiratory state, performed separately for each subject.

The beat-by-beat measures (HR, CO, SV, PEP, LVET, TPR) were interpolated and downsampled to 1 Hz so that each 195 s CPT/WPT trial for every subject consisted of 195 data points. The MAP and HF data were also downsampled to 1 Hz. These 1 Hz data were then used for all subsequent plotting and statistical analyses. Thirty-three participants had complete ECG/ICG data so these were included in the HR, LVET, PEP, CO and SV measures. Three participants were removed from BP and TPR analyses due to missing BP data (due to technical issues) in one or more sessions. Two participants were removed from the HF HRV analyses due to excessive noise in the data.

#### 2.5.2 Pupillometry

Pupil diameter measures were averaged across both eyes and downsampled from 500 Hz to 1 Hz for plotting and statistical analyses and smoothed for visualization purposes using a 5 s moving window. Visual inspection of the data revealed that 22 participants’ data consisted of one or more trials where the eye-tracker failed to detect the eye for one or more trials. These participants were removed and data from the remaining 21 participants were submitted to the same analyses as described for the cardiovascular data (see Section 2.5.1). Given that pupillometry data are recorded in arbitrary units, all data were normalized between 0–1.

#### 2.5.3 EEG

MATLAB (version 2019a, The MathWorks, Inc., Natick, MA) was used for all data processing. The EEGLAB toolbox (v2019.1) (Delorme and Makeig, 2004) was used for offline processing of the EEG data. Data from the five exposures within each session were merged to form a single file for each session, and the data were high-pass filtered at 1 Hz using a trapezoidal filter to remove low-frequency drifts and downsampled to 250 Hz. Line noise (∼60 Hz) was removed with a notch filter and abnormal scalp electrodes were identified whereby a channel was considered to be abnormal if it flatlined for more than 5 seconds, had values exceeding four standard deviations of the total channel population, or failed to correlate at *r* > 0.85 with a reconstruction of it based on other channels. The data were referenced to an average of the scalp channels that excluded the abnormal channels, and then abnormal channels were interpolated back into the dataset with spherical interpolation. Data from 8 participants were removed due to excessive noise, technical issues or missing data, leaving data from 34 participants.

Next, signal processing techniques were used to isolate brain activity from other non-brain sources of electrical noise, so that any effects of the cold vs. warm water manipulation could be directly attributed to changes in brain activity rather than sources of non-brain noise. This approach was necessary because discomfort caused by the CPT resulted in numerous sources of electromyographic (EMG) activity (e.g. jaw clenching, tensing of muscles in face and neck, shivering, fidgeting) as well as ocular artifacts (e.g., blinking, eye closure), and these responses can cause large electrical potentials at frequencies that overlap with brain frequencies of interest. To address the noise contamination, the data were submitted to an adaptive mixture independent components analysis (AMICA; *runamica15.m*) and then used an automated routine to identify ICs that likely represented sources of brain activity. First, an automatic independent component (IC) classifier (*ICLabel.m;* Pion-Tonachini, Kreutz-Delgado, & Makeig, 2019) was used to identify “brain” ICs. ICLabel assigns each IC a classification score in seven different categories (*brain, ocular, EMG, EKG, channel noise, line noise, other*). Next, equivalent dipole source localization of ICs was computed using the DIPFIT plugin (*pop_multifit.m*). IC rejection was then implemented using ICLabel and dipole information, such that an IC was only accepted if it 1) was classified as 70% or above in the “brain” category, 2) contributed <15% residual variance of the IC scalp maps, and 3) the dipole was located inside the brain. All other components were excluded and the data were reconstructed based on the accepted ICs.

Event-related spectral perturbations (ERSPs) were then computed to assess stress-evoked changes across the range of frequency bands typically associated with cognitive function (i.e., ∼1–30 Hz). ERSPs had a 1 Hz frequency resolution, 1 Hz temporal resolution and 4 Hz moving window (*newtimef.m*).

#### 2.5.4 Saliva

Saliva samples were collected throughout each experiment session in individual 3.6 ml Cryogenic Tubes (ThermoFisher Scientific, Waltham, MA, United States) and stored at −80°C until being shipped for analysis. Samples were shipped on dry ice and assayed at the University of California Davis Clinical Endocrinology Lab using standard ELISA cortisol assay kits (Salimetrics, Inc., Carlsbad, CA, United States). Samples were submitted to a single assay and the inter-assay coefficient of variability was 4.93%

### 2.6 Common analytical framework

All data are were analyzed and interpreted under a common framework. First, for each measure, two-way ANOVAs were computed to determine whether there were any main effects of condition, habituation across trials, and any condition x trial interactions. Significant interactions were then explored by computing pairwise comparisons to investigate intra-session habituation across trials. First, T1 and T5 are compared, to determine whether the stress-response habituates over the entire session. Second, to gain a sense of whether these habituation effects are driven by habituation across earlier or later exposures within the session, T1 and T3 are compared (early-session habituation) and T3 and T5 are compared (late-session habituation). All statistical tests were conducted using a non-parametric permutation-based resampling approach (Section 2.7).

For cardiovascular and pupillometry measures specifically, the appropriate tests are computed for each second of trial data, to gain insight into the time course of intra-trial stress and habituation dynamics. For the time-course analyses, ANOVA and *t*-test outcomes where *p*_null_<0.05 are represented by the presence of colored horizontal bars superimposed onto the plots, where the presence of a bar at a given time point indicates a significant difference. ANOVA outcomes are presented in-between the treatment and control plots, and pairwise comparison outcomes are presented at the base of the treatment and control plots for timepoints where a significant condition x trial interaction is observed, where each color pair represents a different comparison i.e., red/blue = T1 vs. T5 (habituation across entire session), red/yellow = T1 vs. T3 (early -session habituation), yellow/blue = T3 vs. T5 (late-session habituation). The goal of analyzing the raw data was to study stress effects that occurred *prior to* foot immersion (i.e., 0–65 s), hence the “immersion” and “recovery” periods are greyed out in the raw plots and statistical comparisions are not reported for these time periods. The goal of analyzing the baseline corrected data was to study stress effects *evoked by* foot immersion (i.e., 65 s–195 s), hence the “baseline” and “prep” periods are greyed out in the baseline corrected plots and statistical comparisions are not reported for these time periods.

To address whether a change occurred *within* either condition for all the cardiovascular variables we computed additional three-way ANOVA analyses on the raw data that incorporated change in baseline as a factor, comparing data recorded during the pre-CPT baseline sessions with data recorded during and after the CPT exposures. In our main baseline-corrected analyses we computed baseline by averaging over the final 15 s of the baseline period, so for consistency we also applied a 15 s moving window average to the data recorded during and after the CPT (i.e. 65-155 s). We then computed a 2 [baseline: pre-CPT, during/post CPT] x 2 [condition: treatment, control] x 5 [trial: T1>T5] ANOVA for all timepoints ranging from 65–140 s. Consistent with our main analyses, the complete results from the ANOVAs are plotted with the horizontal bars between the figures representing significant main and interaction effects (Supplementary Figure S1).

For the EEG data, baseline uncorrected ERSP data are presented averaged across the baseline period (1–40 s) and baseline corrected ERSP data averaged across early (65–94 s), mid (95–124 s), late (125–154 s) and recovery (155–190 s) periods. As before, inspection of the baseline uncorrected data provides insight into any potential changes in brain oscillations that occur prior to exposure, and inspection of baseline corrected data allows us to look at changes *evoked* by exposure. The data were also parsed into different frequency bands associated with cognitive function i.e. delta [1–3 Hz], theta [4–7 Hz], alpha [8–14 Hz] and beta [15–30 Hz]. A 2 [stress condition] x 5 [trial] ANOVA was then computed for each scalp electrode for each time period and frequency band, and any robust condition x trial interactions were computed by averaging across all electrodes that contribute to the interaction and computing pairwise comparisons as described above.

### 2.7 Hypothesis testing

Statistical significance in all tests (except the rank order analysis) was assessed by using a non-parametric permutation-based resampling approach to empirically approximate null distributions for *F* and *t* statistics (Foster et al., 2016; Bullock et al., 2017; Bullock et al., 2021). This testing approach was adopted because it has the advantage of being robust to violations of normality. Specifically, condition labels were shuffled within participants and 1000 iterations of the appropriate statistical tests were ran (repeated-measures ANOVAs and/or paired-samples *t* tests) to generate null distributions of *F* and *t* statistics. Once null distributions had been obtained, reliable differences were tested for by computing the probability of obtaining *F* and *t* statistics from each of the null distributions that were greater than the observed *F* and *t* statistics. The *F* and *t* statistics are reported in the text along with the critical *p*-value (labelled *p*_null_) which represents the probability of observing a value greater than this in the null distribution. For the time-course data analyses (Cardiovascular Measures, Pupillometry) ANOVA and *t*-test outcomes where *p*_null_<0.05 are represented by the presence of colored horizontal bars superimposed onto the plots, where the presence of a bar at a given time point indicates a significant difference. ANOVA outcomes are presented in-between the treatment and control plots, and pairwise comparisons are presented at the base of the treatment and control plots for timepoints where a significant condition x trial interaction is observed (see Section 3.2 for detailed description). For self-report, EEG and cortisol analyses, test results are reported in the text. Here, to gain a more precise estimate of the observed statistic’s position in the null distribution, tests are reported as *p*_null_<0.05, *p*_null_<0.01 and *p*_null_<0.001. A test result of *p*_null_>0.05 indicates that the result was not statistically reliable. The time-course analyses presented here rely on repeated comparisons at multiple timepoints, which raises the possibility of increased inferential error, however, the effects that are described are present across multiple timepoints and no inference relies on a single comparison but rather a consistent pattern across time.

### 2.8 Data availability

All custom analysis scripts are available *via* GitHub (https://github.com/attlab/CPT_Adaptation). Data are available upon request.

## 3 Results

### 3.1 Self-reported pain ratings

**FIGURE 3:**
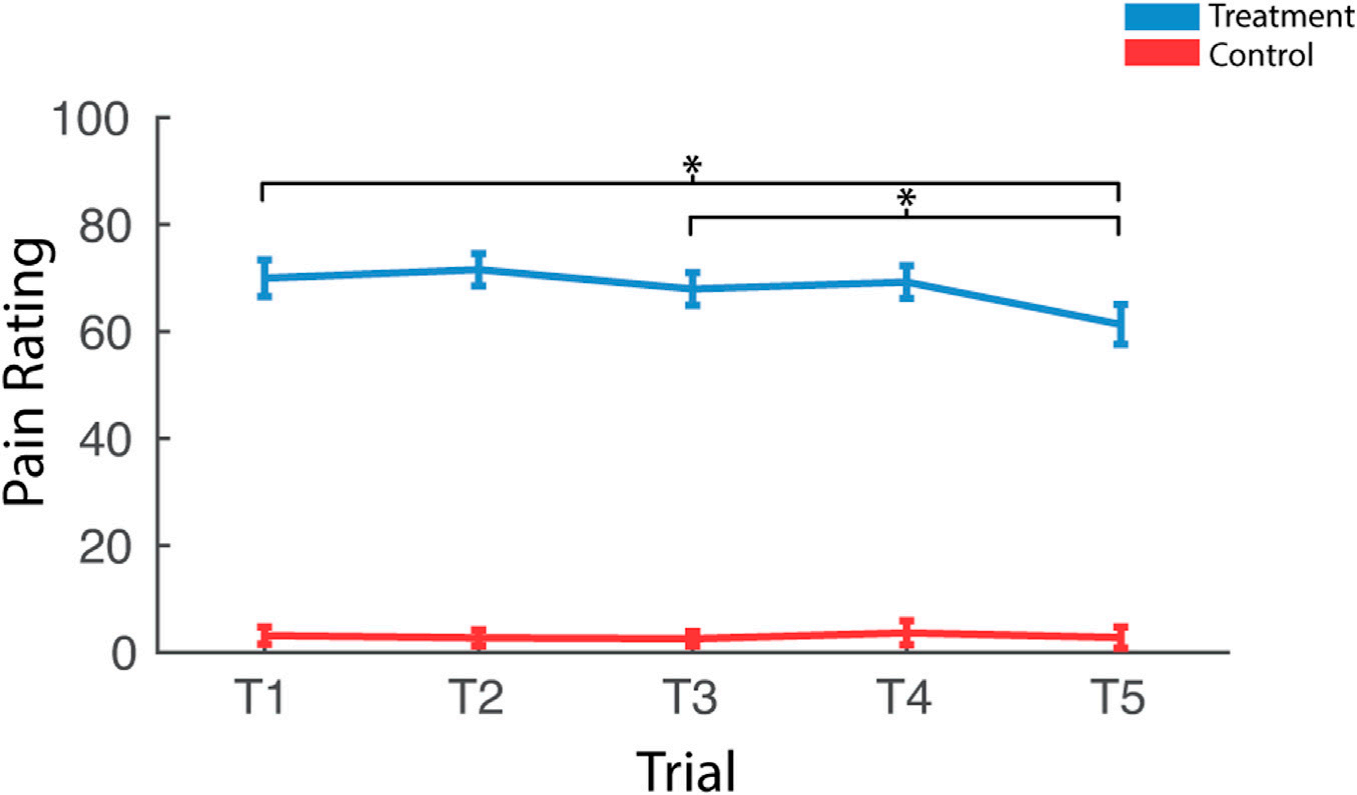
Effects of treatment and repeated trial exposure on self-reported pain. Participants were asked to provide pain ratings at the termination of each treatment or control trial. *p_null_<0.05. Error bars = ± SEM.

Participants reported significantly greater pain immediately after treatment trials when compared to control trials [*F* (1,37) = 392.54, *p*_null_<0.001, *η*_*p*_^*2*^ = 0.91] (Figure 3). There was also an interaction between condition and trial [*F* (4,148) = 8.06, *p*_null_<0.001, *η*_*p*_^*2*^ = 0.18]. Pairwise comparisons revealed that this interaction was driven by reduced pain ratings as a function to repeated exposures over the course of the treatment session [T1>T5; t (37) = 3.41, *p*_null_<0.001] and this was driven by late-session habituation [T3>T5; t (37) = 3.33, *p*_null_<0.001] not early-session habituation [T1>T3; t (37) = 1.09, *p*_null_>0.05]. There was very little variability in pain ratings in control trials, with most participants rating all five trials as inducing no pain (rating = 0), so pairwise comparisons were not computed for control trials.

### 3.2 Cardiovascular and pupillometry measures

All cardiovascular and pupillometry measures are presented in both raw and baseline corrected format and analyzed using a common framework (see Section 2.6). Resting baseline levels for all cardiovascular measures are reported in Table 1. Note that these resting recordings were taken from the final 15 s of the baseline period in the first control exposure (i.e., lukewarm water) which. We reason that participants were in a relatively relaxed state at this time given that they were explicitly aware the forthcoming exposure was to lukewarm water; however, it is possible that anticipation of the exposure and elevated stress levels from being in the lab and having sensors applied to the face and body may be associated with elevated ABP and increased physiological arousal. Hence, these values may deviate from a true resting baseline.

#### 3.2.1 Heartbeat rate and variability

##### 3.2.1.1 HR pre-exposure

**FIGURE 4:**
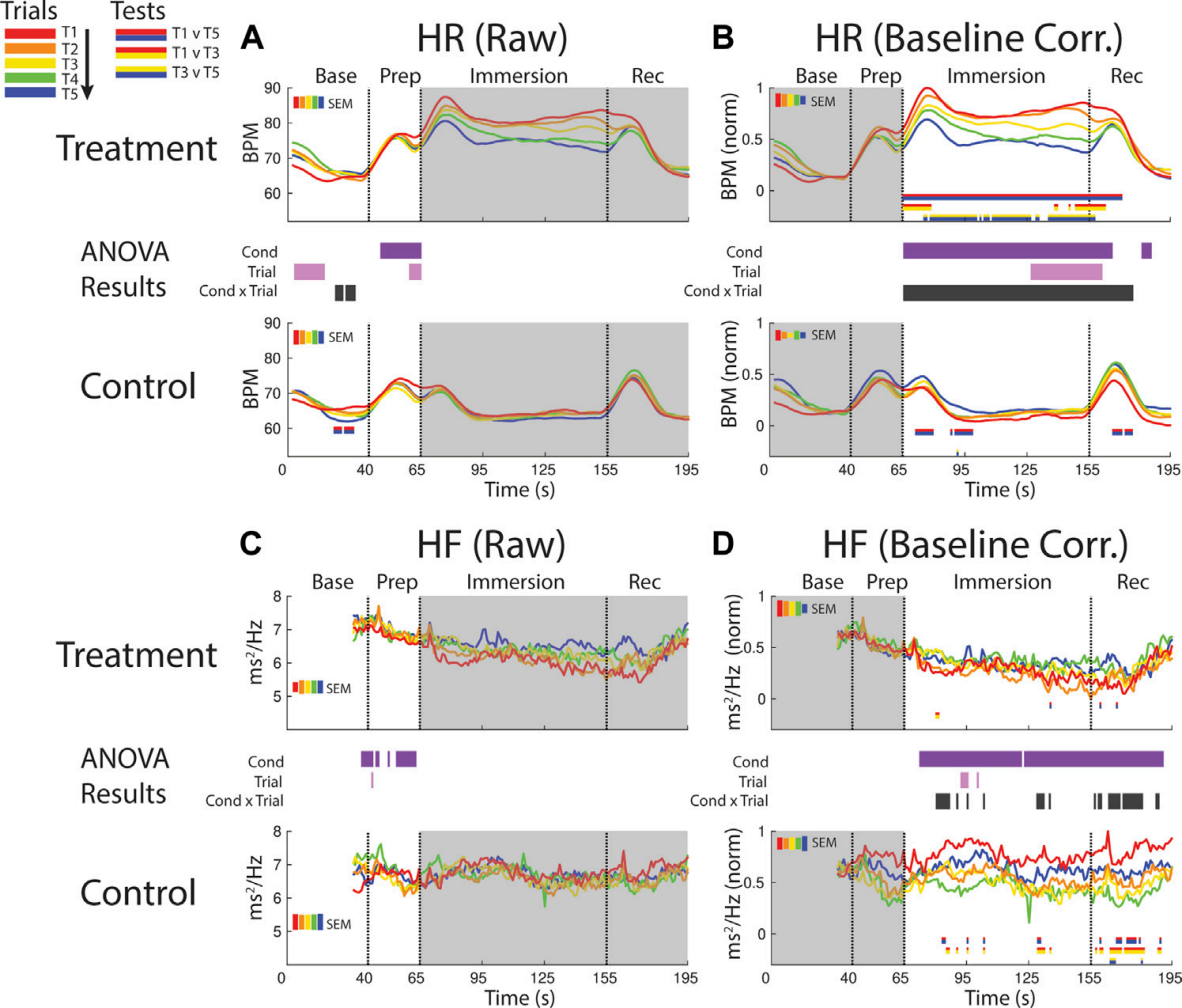
Effects of treatment and repeated trial exposure on heartbeat rate [HR; **(A**,**B)**] and high frequency HR variability [HF; **(C**,**D)**]. Pre-exposure and evoked stress effects are reflected in raw (left column) and baseline corrected/normalized (right column) plots, respectively. Each panel contains a separate plot for the treatment and control condition, with the mean response across participants in each repetition trial (T1-T5) represented in each plot by a different color (ROYGB). Horizontal lines positioned between treatment and control plots indicate statistically significant ANOVA main effects of condition (dark purple), trial (light purple) and the condition x trial interaction (dark gray) at designated timepoints (*p*_null_<0.05). Horizontal red/blue, red/yellow and yellow/blue lines at the base of treatment and control plots respectively indicate statistically significant pairwise comparison results for T1 v T5, T1 v T3 and T3 v T5 at the designated timepoints (*p*_null_<0.05). Post-immersion raw data and pre-immersion baseline corrected normalized data are greyed out as these periods are not relevant to the pre-exposure/evoked stress analyses. The set of five vertical error bars labelled SEM on each plot represent ±SEM of the data averaged over the time-course of interest.

HR measured during the early baseline period increased over the course of the session, supported by a main effect of trial. HR habituated in the late baseline period in control, declining over the course of the session, supported by a condition x trial interaction and T1 v T5 pairwise comparisions (Figure 4A).

##### 3.2.1.2 HR evoked

HR was elevated throughout immersion during the treatment condition when compared to the control condition, verified by a main effect of condition throughout immersion. HR also habituated across trials, supported by a condition x trial interaction present throughout immersion and T1 v T5 pairwise effects in treatment. This interaction during treatment was initially driven by early session habituation differences (T1>T3), then by late-session habituation (T3>T5) and then by both. HR then briefly surged in the recovery period immediately after participants removed their feet from the water bucket, but only in control trials and late-session treatment trials (T3-T5), before declining back towards baseline (Figure 4B).

##### 3.2.1.3 HF pre-exposure

It is not possible to analyze the initial 32 s of the baseline period as these data are used in the HF modeling. However, inspection of the final seconds of the baseline period and the prep period in the raw data reveals higher HF during treatment when compared to control, supported by a main effect of condition (Figure 4C).

##### 3.2.1.4 HF evoked

Inspection of the baseline corrected data revealed a drop in HF in the immersion period during treatment when compared to control, indicating a decline in HF as a function of cold-water exposure, supported by a main effect of condition. There is some sporadic evidence for habituation during immersion and recovery, supported at some timepoints by the condition x trial interaction and generally driven by early session habituation (T1>T3) in control (Figure 4D).

#### 3.2.2 Sympathetic drive and cardiac timing

##### 3.2.2.1 PEP pre-exposure

**FIGURE 5:**
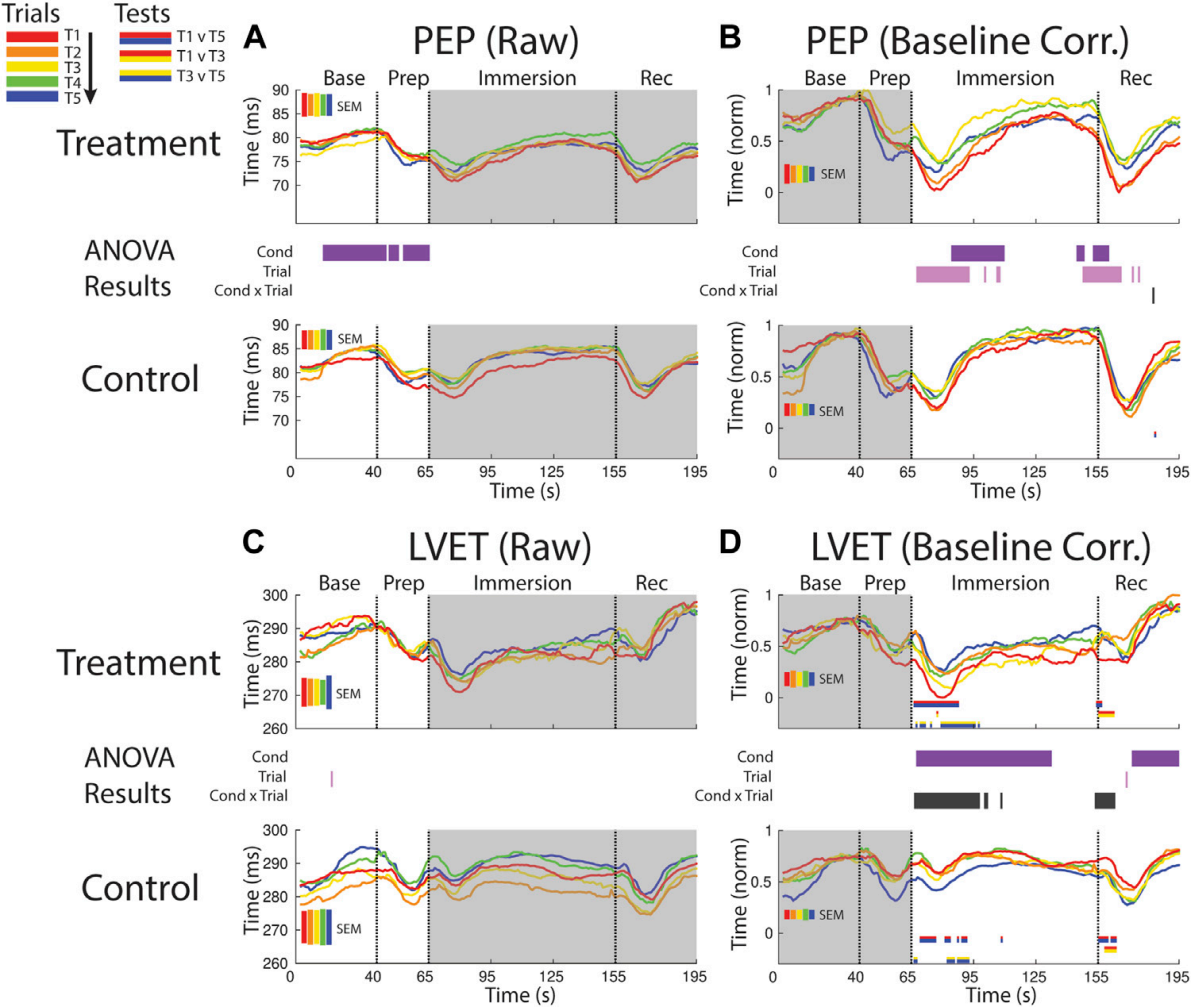
Effects of treatment and repeated trial exposure on sympathetic drive [PEP; **(A**,**B)]** and cardiac timing [LVET; **(C**,**D)]**. Pre-exposure and evoked stress effects are reflected in raw (left column) and baseline corrected/normalized (right column) plots, respectively. Each panel contains a separate plot for the treatment and control condition, with the mean response across participants in each repetition trial (T1-T5) represented in each plot by a different color (ROYGB). Horizontal lines positioned between treatment and control plots indicate statistically significant ANOVA main effects of condition (dark purple), trial (light purple) and the condition x trial interaction (dark gray) at designated timepoints (*p*_null_<0.05). Horizontal red/blue, red/yellow and yellow/blue lines at the base of treatment and control plots respectively indicate statistically significant pairwise comparison results for T1 v T5, T1 v T3 and T3 v T5 at the designated timepoints (*p*_null_<0.05). Post-immersion raw data and pre-immersion baseline corrected normalized data are greyed out as these periods are not relevant to the pre-exposure/evoked stress analyses. The set of five vertical error bars labelled SEM on each plot represent ±SEM of the data averaged over the time-course of interest.

A decline in PEP (increased sympathetic drive) was observed during baseline and prep periods (∼15–65 s), driven by lower PEP in the treatment condition when compared to the control condition. This was supported by a main effect of condition (Figure 5A).

##### 3.2.2.2 PEP evoked

PEP declined immediately after foot immersion in both treatment and control conditions but then began to return to baseline at a faster rate during control relative to treatment, supported by a main effect of condition (∼80–110 s). PEP also habituated in both conditions over the course of the study. This habituation occurred in the immersion period (∼70–110 s) and leading into the recovery period, supported by a main effect of trial (Figure 5B).

##### 3.2.2.3 LVET pre-exposure

LVET trended upwards in both treatment and control sessions during the baseline periods before declining in the prep period, but no significant differences between conditions or exposures were reported (Figure 5C).

##### 3.2.2.4 LVET evoked

LVET was lower throughout the majority of the immersion and recovery periods during treatment when compared to control, supported by a main effect of condition (∼70–135 s; ∼160–195 s, respectively). LVET also habituated at the start of the immersion and recovery periods, supported by a condition x trial interaction (∼65–100 s; ∼155–160 s, respectively). These interactions were driven by a reduction in the LVET response to immersion during treatment (T1>T5 and T3>T5) and also a drop in LVET during the control condition (T1>T5 and T3>T5; Figure 5D).

#### 3.2.3 Cardiac output and volume

##### 3.2.3.1 CO pre-exposure

**FIGURE 6:**
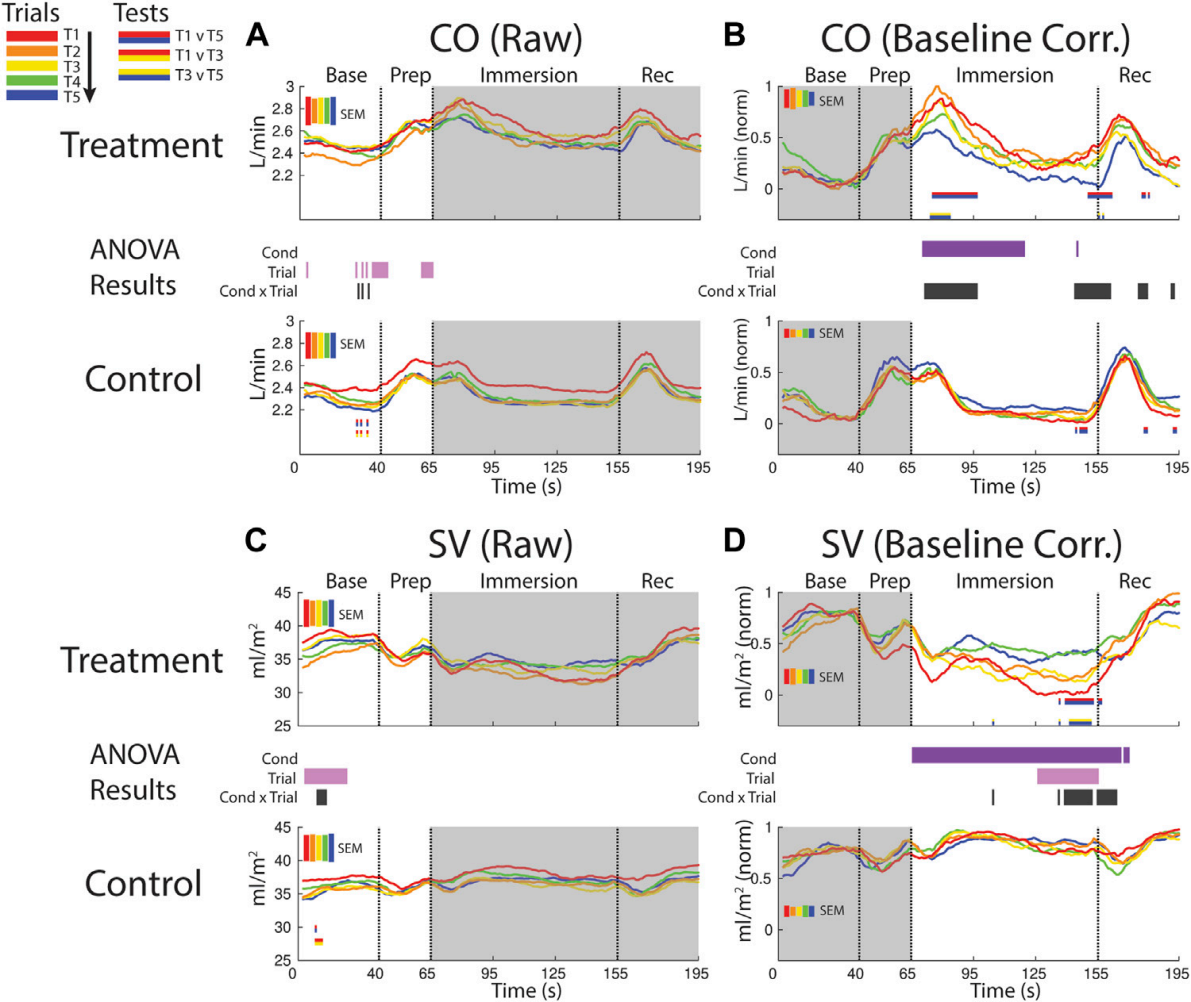
Effects of treatment and repeated trial exposure on cardiac output [CO; **(A**,**B)]** and stroke volume [SV; **(C**,**D)]**. Pre-exposure and evoked stress effects are reflected in raw (left column) and baseline corrected/normalized (right column) plots, respectively. Each panel contains a separate plot for the treatment and control condition, with the mean response across participants in each repetition trial (T1-T5) represented in each plot by a different color (ROYGB). Horizontal lines positioned between treatment and control plots indicate statistically significant ANOVA main effects of condition (dark purple), trial (light purple) and the condition x trial interaction (dark gray) at designated timepoints (*p*_null_<0.05). Horizontal red/blue, red/yellow and yellow/blue lines at the base of treatment and control plots respectively indicate statistically significant pairwise comparison results for T1 v T5, T1 v T3 and T3 v T5 at the designated timepoints (*p*_null_<0.05). Post-immersion raw data and pre-immersion baseline corrected normalized data are greyed out as these periods are not relevant to the pre-exposure/evoked stress analyses. The set of five vertical error bars labelled SEM on each plot represent ±SEM of the data averaged over the time-course of interest.

CO habituated across trials in the baseline and prep periods, supported by a main effect of trial. A condition x trial interaction also fleetingly emerged, with pairwise comparisions suggesting the control condition selectively habituated during the baseline period, but this effect should be interpreted with caution given it is only present for a small number of timepoints (Figure 6A).

##### 3.2.3.2 CO evoked

CO increased and remained elevated during the first part of the immersion period in the treatment condition relative to control, supported by a main effect of condition (∼70–115 s). CO also habituated early in the immersion period and also towards the end of immersion entering into recovery. This was supported by a condition x trial interaction (∼70–95 s; ∼140–160 s) and mainly T1 v T5 pairwise tests in the treatment condition (Figure 6B).

##### 3.2.3.3 SV pre-exposure

SV declined in the baseline period during both treatment and control sessions, supported by a trial effect (∼5–25 s). SV also habituated across control trials, supported by a condition x trial interaction and primarily T1 v T3 pairwise tests (Figure 6C).

##### 3.2.3.4 SV evoked

SV was consistently lower throughout the immersion period during treatment when compared to control, supported by a condition effect. There was also evidence for early-session habituation towards the end of the immersion period and very beginning of recovery, verified by a condition x trial interaction and T1 v T5 and T3 v T5 pairwise tests in treatment (∼135–160 s; Figure 6D).

#### 3.2.4 MAP and TPR

##### 3.2.4.1 MAP pre-exposure

**FIGURE 7:**
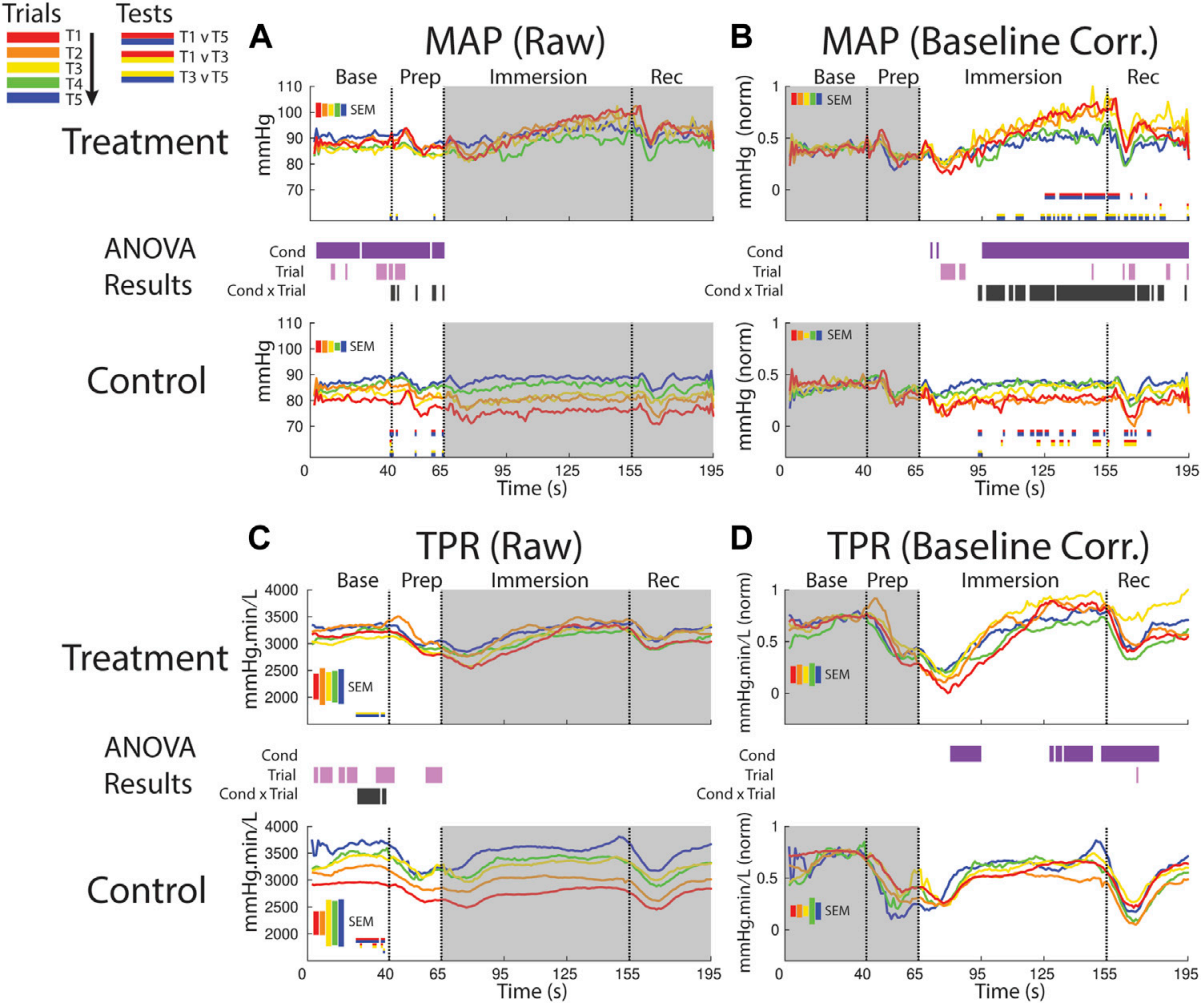
Effects of treatment and repeated trial exposure on mean arterial pressure [MAP; **(A**,**B)]** and total peripheral resistance [TPR; **(C**,**D)]**. Pre-exposure and evoked stress effects are reflected in raw (left column) and baseline corrected/normalized (right column) plots, respectively. Each panel contains a separate plot for the treatment and control condition, with the mean response across participants in each repetition trial (T1-T5) represented in each plot by a different color (ROYGB). Horizontal lines positioned between treatment and control plots indicate statistically significant ANOVA main effects of condition (dark purple), trial (light purple) and the condition x trial interaction (dark gray) at designated timepoints (*p*_null_<0.05). Horizontal red/blue, red/yellow and yellow/blue lines at the base of treatment and control plots respectively indicate statistically significant pairwise comparison results for T1 v T5, T1 v T3 and T3 v T5 at the designated timepoints (*p*_null_<0.05). Post-immersion raw data and pre-immersion baseline corrected normalized data are greyed out as these periods are not relevant to the pre-exposure/evoked stress analyses. The set of five vertical error bars labelled SEM on each plot represent ±SEM of the data averaged over the time-course of interest.

Mean arterial blood pressure (MAP) was consistently elevated during baseline and prep during treatment when compared to control, supported by a main effect of condition. MAP also increased over both sessions, as verified by a main effect of trial (∼35–45 s). There was also some evidence for anticipatory stress habituation, driven by changes in the control session during prep (∼40–65 s) but the supporting interaction and pairwise tests were only significant at sporadic timepoints in this window (Figure 7A).

##### 3.2.4.2 MAP evoked

MAP increased during the treatment session relative to the control session beginning ∼30 s into the immersion period, supported by a main effect of condition. Late-session habituation in treatment began approximately one-third of the way through immersion (∼95 s), supported by a condition x trial interaction. This habituation was driven by a reduced increase in ABP in T4 & 5 compared to T1-3, supported by T1 v T5 (∼125–160 s) and T1 v T3 (sporadic, ∼100–195 s) pairwise comparisons. Furthermore, while MAP is generally lower in control than treatment trials throughout most of the immersion period, MAP in the control session increased from earlier to later trials i.e. repeated exposure to warm water increased MAP, as supported by T1 v T5 and T1 v T3 pairwise tests (sporadic, ∼95–175 s; Figure 7B).

##### 3.2.4.3 TPR pre-exposure

TPR increased over the course of the session in both conditions in the baseline and prep periods, supported by a main effect of trial (several blocks of time between 1–65)*.* Habituation also was observed in TPR in the baseline period, supported by an interaction (∼25–45 s) and driven by changes in both treatment (pairwise tests indicate increased TPR from T3 > T5) and control (pairwise tests indicate increased TPR from T1 > T5; Figure 7C).

##### 3.2.4.4 TPR evoked

Following immersion TPR declined and was lower in the treatment condition relative to the control condition; however, TRP then recovered in the treatment condition and increased relative to control during the final ∼30 s of immersion and into the recovery period. These effects are supported by a main effect of condition (∼80–95 s and 130–180 s; Figure 7D).

#### 3.2.5 Within condition analyses

The results of the cardiovascular data 3-WAY ANOVA analyses that incorporated change in baseline as an additional factor are presented in Supplementary Figure S1. The primary aim of these analyses were to determine whether each measure shifts away from baseline as a function of both cold and warm water exposure. All cardiovascular variables did shift away from baseline in response to immersion in both treatment and control conditions, supported by a main effect of baseline in each case. This shift started immediately after immersion and was sustained throughout immersion is for all measures except PEP, TPR and MAP, where it disappears mid-immersion then returns later. Critically, for each measure, a baseline x condition and/or baseline x condition x trial interaction also emerged at some point during immersion, indicating that while there may be a baseline shift, there is also a difference in the responses to cold and warm water. For completeness, all other main and interaction effects are also shown but these are not discussed.

### 3.3 Pupillometry

#### 3.3.1 Pupil pre-exposure

**FIGURE 8:**
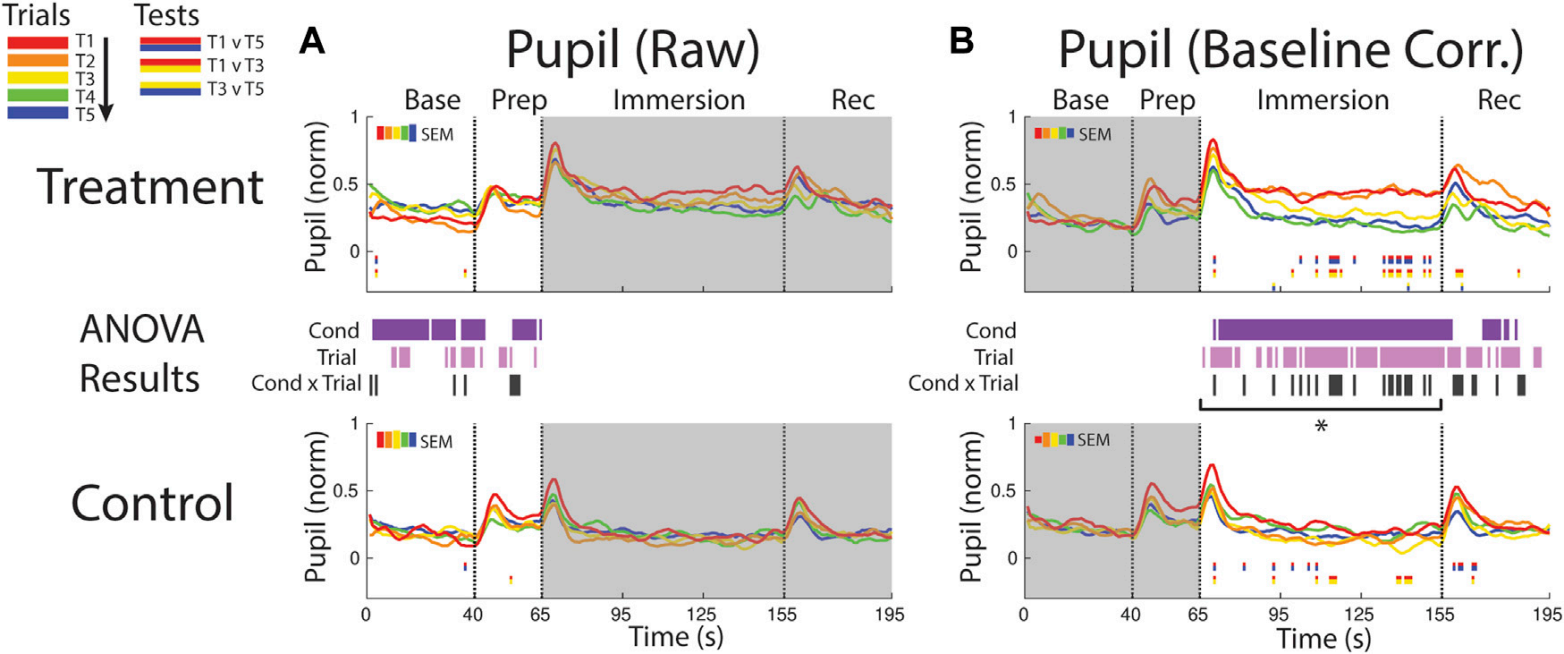
Effects of treatment and repeated trial exposure on pupil size. Pre-exposure and evoked stress effects are reflected in **(A)** raw and **(B)** baseline corrected/normalized plots, respectively. Each panel contains a separate plot for the treatment and control condition, with the mean response across participants in each repetition trial (T1-T5) represented in each plot by a different color (ROYGB). Horizontal lines positioned between treatment and control plots indicate statistically significant ANOVA main effects of condition (dark purple), trial (light purple) and the condition x trial interaction (dark gray) at designated timepoints (*p*_null_<0.05). Horizontal red/blue, red/yellow and yellow/blue lines at the base of treatment and control plots respectively indicate statistically significant pairwise comparison results for T1 v T5, T1 v T3 and T3 v T5 at the designated timepoints (*p*_null_<0.05). Post-immersion raw data and pre-immersion baseline corrected normalized data are greyed out as these periods are not relevant to the pre-exposure/evoked stress analyses. The set of five vertical error bars labelled SEM on each plot represent ±SEM of the data averaged over the time-course of interest. * Due to technical issues with eye-tracking, this pupillometry analysis was underpowered relative to analyses of the other measures, so the results of the timepoint-by-timepoint analysis are not definitive. Accordingly, the data were averaged across the immersion time period and submitted to an additional ANOVA, revealing a robust trial x condition interaction, driven by early-session habituation.

The pupil was generally more dilated in the baseline period and later in the prep period during treatment when compared to control (supported by a condition effect) suggesting persistent anticipatory stress effects during the treatment session (Figure 8A). There was some evidence for increased pupil size over the course of the session, but the supporting trial effect was only observed at sporadic timepoints during baseline and prep periods.

#### 3.3.2 Pupil evoked

Pupil size increased in both conditions immediately after the instruction to immerse both feet in water (65 s), but the increase was larger and sustained throughout the immersion period in treatment trials, confirmed with a condition effect (∼70–155 s and ∼165–180 s). The point-by-point ANOVA and pairwise results suggested there was some evidence for early-session habituation approximately midway through the immersion period during treatment, but the results were not robust, likely due to the reduced sample size (*n* = 21) in this analysis that resulted from lost eye-tracking data (Figure 8B). Given that this analysis was underpowered relative to other reported measures, an additional set of analyses were computed where the data were first averaged across the immersion period (66–155 s) to reduce noise and then submitted to an ANOVA and pairwise comparisions. Consistent with the point-by-point results, main effects of condition [*F* (1,20) = 24.65, *p*_null_<0.001] and trial [*F* (4,80) = 7.32, *p*_null_<0.001] were observed as well as a robust interaction [*F* (4,80) = 4.28, *p*_null_<0.001]. Pairwise comparisions revealed a significant reduction in pupil diameter over the treatment session [T1>T5: [*t* (20) = 3.49, *p*_null_<0.001], driven by early-session habituation [T1>T3: *t* (20) = 2.90, *p*_null_<0.05]. Early-session habituation was also observed in the control session [T1>T3: *t* (20) = 2.70, *p*_null_<0.05]. All other comparisons were non-significant (*p*_null_>0.05).

### 3.4 Oscillatory brain activity

**FIGURE 9:**
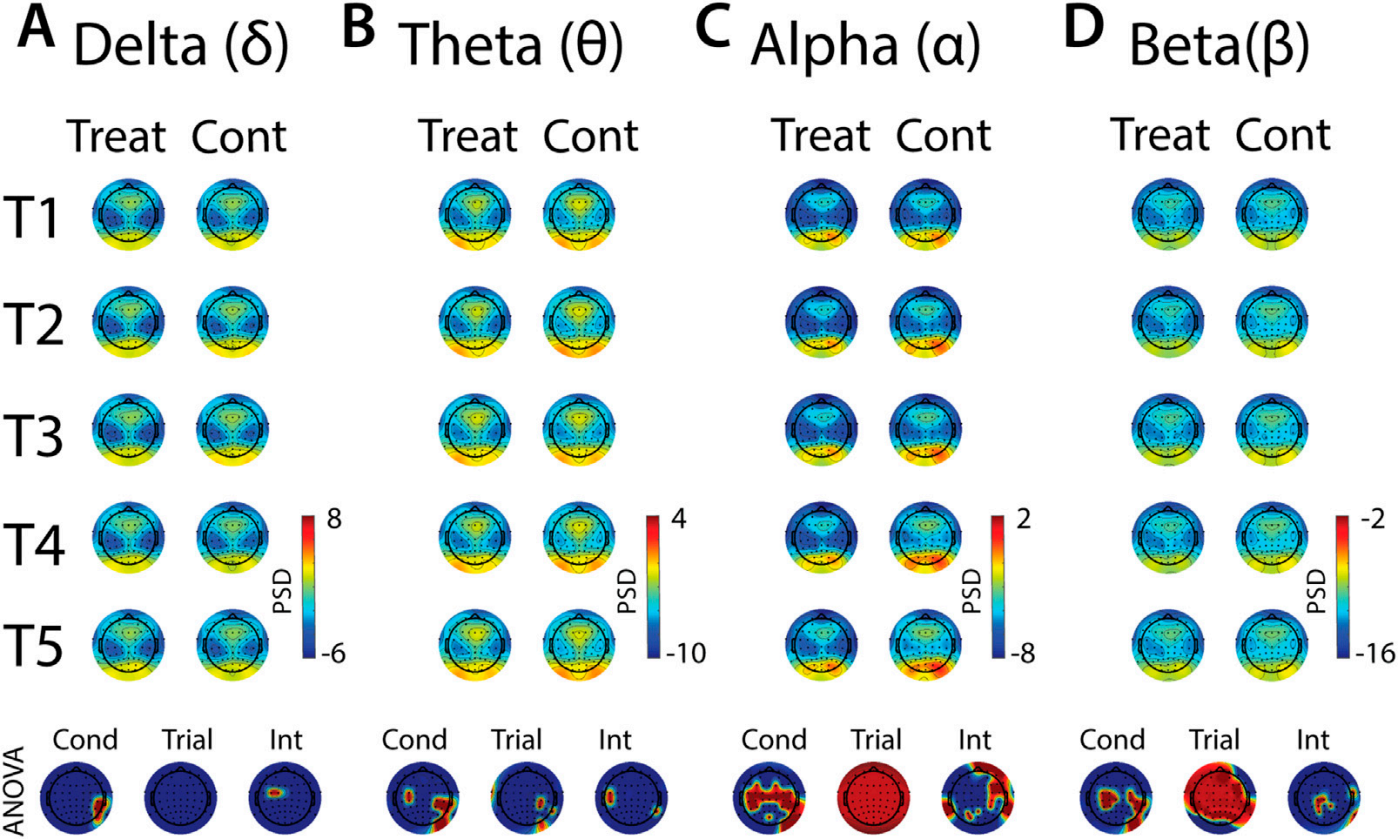
Effects of treatment and repeated trial exposure on anticipatory neural activity. Topographic plots in each panel **(A**–**D)** depict pre-exposure oscillatory neural activity in the delta, theta, alpha and beta frequency bands, respectively. Each panel contains separate columns of plots for the treatment and control conditions, with each repetition trial (T1-T5) represented by each row. Each plot depicts brain activity averaged across the baseline period. Condition (treatment, control) by trial (T1-T5) ANOVA results at each electrode are plotted at the base of each panel, with condition, trial and interaction effects depicted in separate topographic plots. Scalp regions overlaid in red indicate significant effects (*p*_null_<0.05).

**FIGURE 10:**
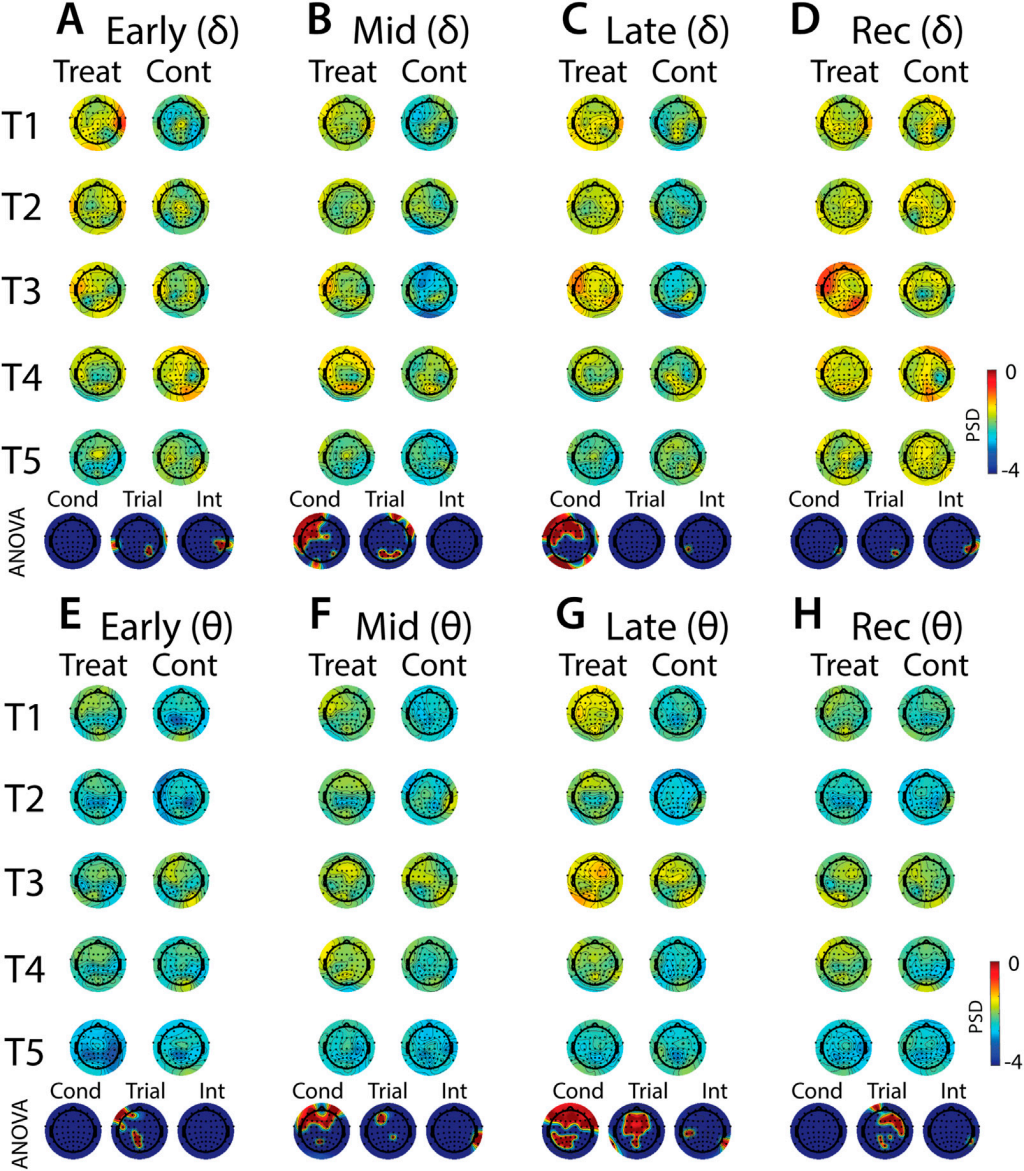
Effects of treatment and repeated trial exposure on stress-evoked oscillatory neural activity in delta and theta frequency bands. Topographic plots in each panel depict evoked stress effects on oscillatory neural activity in the delta **(A**–**D)** and theta **(E**–**H)** frequency bands. Topographic plots in panels **A**–**D** and **E**–**H** respectively represent brain activity averaged across the early (65–94 s), mid (95–124 s), late (125–154 s) and recovery (155–190 s) periods of the immersion period. Each panel contains separate columns of plots for the treatment and control conditions, with each repetition trial (T1-T5) represented by each row. Condition (treatment, control) by trial (T1-T5) ANOVA results at each electrode are plotted at the base of each panel, with condition, trial and interaction effects depicted in separate topographic plots. Scalp regions overlaid in red indicate significant effects (*p*_null_<0.05).

**FIGURE 11:**
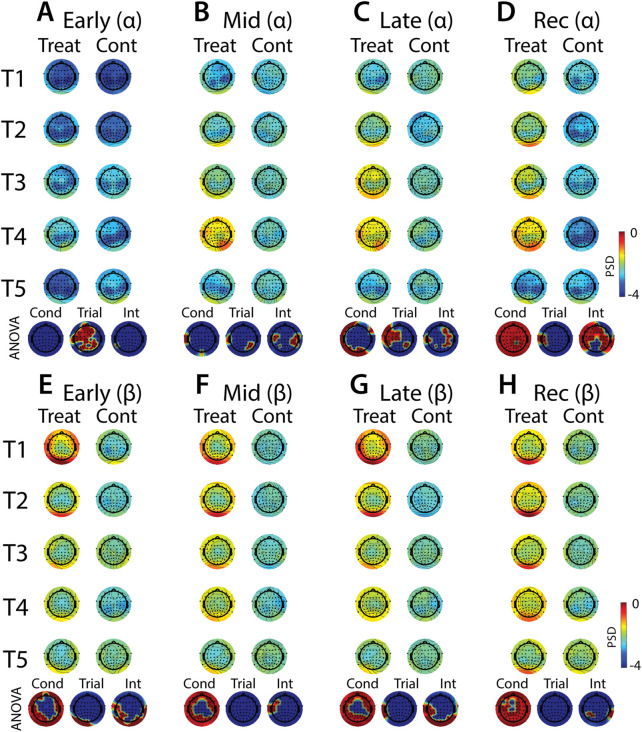
Effects of treatment and repeated trial exposure on stress-evoked oscillatory neural activity in alpha and beta frequency bands. Topographic plots in each panel depict evoked stress effects on oscillatory neural activity in the alpha **(A**–**D)** and beta **(E**–**H)** frequency bands. Topographic plots in panels **A**–**D** and **E**–**H** respectively represent brain activity averaged across the early (65–94 s), mid (95–124 s), late (125–154 s) and recovery (155–190 s) periods of the immersion period. Each panel contains separate columns of plots for the treatment and control conditions, with each repetition trial (T1-T5) represented by each row. Condition (treatment, control) by trial (T1-T5) ANOVA results at each electrode are plotted at the base of each panel, with condition, trial and interaction effects depicted in separate topographic plots. Scalp regions overlaid in red indicate significant effects (*p*_null_<0.05).

Analyses of anticipatory and evoked stress effects on neural oscillations are presented in Figures 9–11. A common analytical framework was applied to the EEG data that was similar to the framework applied to cardiovascular and pupillometry data (see Section 2.6 for a detailed account), but for data reduction purposes the EEG data are parsed into key frequency bands (delta, theta, alpha, beta) and time periods (baseline, early-immersion, mid-immersion, late-immersion and recovery).

#### 3.4.1 EEG pre-exposure

In all frequency bands, modulation of activity in right posterior-temporal scalp locations was observed, supported by main effects of condition (Figures 9A–D). Theta also increased across bilateral central electrodes in anticipation of cold-water, but the activation was less robust in the left hemisphere (Figure 9B). Robust condition effects were observed in alpha and beta bands, driven by lower alpha and higher beta power across bilateral central electrode sites in anticipation of cold-water exposure relative to warm-water exposure (Figures 9C, D). Global alpha and beta power also increased over the course of the five trials in both treatment and control conditions. Interaction effects were present in all frequency bands, but these effects were either present in just a few diffuse electrode locations or were difficult to interpret given their topographical distribution. Together, these results indicate that anticipation of cold is observed over motor sites in both alpha and beta and does not evolve over multiple exposures, and that there are no other strong interpretable effects of anticipatory habituation to cold-water in the brain.

#### 3.4.2 EEG evoked stress

Analyses of the effects of treatment and repeated trial exposure are presented for delta (Figures 10A–D), theta (Figures 10E–H), alpha (Figures 11A–D) and beta (Figures 11E–H) frequency bands. Greater delta and theta activation was observed in fronto-central scalp regions during treatment when compared to control, starting mid-exposure (Figures 10B–D & Figures 10F–H). This activation was left-lateralized in delta but spread across both hemispheres in theta. Midline theta also declined from T1>T5 in both conditions during the late-exposure and recovery periods, suggesting this frequency band’s possible involvement with participants adapting to the water exposures over the course of the trials (Figures 10C, D & Figures 10G, H). Differences between treatment and control sessions were also observed in the alpha and beta frequency bands (Figure 11). Increased activity during treatment was present in mid- and late-exposure periods in alpha (Figures 11B, C) and across all exposure periods in beta (Figures 11E–H), but the topographical distribution of this activation suggests this effect was driven by off-scalp sources of noise rather than by neural activity. Alpha and beta power were also elevated across most of the scalp in the treatment session during the recovery period (Figures 11D, H). A trial x condition interaction was present in alpha during mid, late and recovery periods, with the significance of the interaction effect most widespread during recovery. Data from all electrode sites that contributed to these interactions were averaged and submitted to pairwise comparisons. Tests revealed that changes in the late exposure period were driven by increased alpha from early-to mid-session treatment trials [T1>T3: *t* (33) = −3.54, *p*_null_<0.001] and decreased alpha from mid-to late-session trials [T3<T5: *t* (33) = 3.69, *p*_null_<0.001], whereas changes in the recovery period were driven both by decreased alpha across the whole session [T1>T5: *t* (33) = 2.28, *p*_null_<0.01] and decreased alpha from mid-to late-session [T3>T5: *t* (33) = 3.18, *p*_null_<0.001]. All other pairwise comparisons were non-significant (*p*_null_>0.05).

### 3.5 Cortisol

**FIGURE 12:**
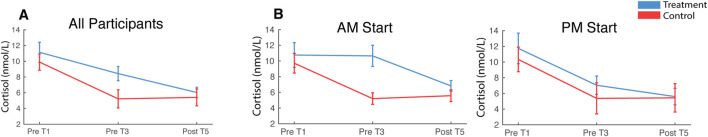
Cortisol. Saliva samples were acquired prior to the first and third pressor tests and at the end of the session. Plots depict cortisol levels averaged across all participants **(A)** and split by participant start time **(B)**. Error bars represent ±SEM.

Cortisol was elevated during treatment relative to the control session [*F* (1,41) = 4.74, *p*_null_<0.05] and declined over the course of the experiment from Sample 1 (Pre T1) to Sample 3 (Post T5) in both conditions [*F* (2,82) = 14.07, *p*_null_<0.001]. Condition and sample did not interact [*F* (2,82) = 2.13, *p*_null_>0.05] (Figure 12A). Circulating cortisol levels fluctuate throughout the day, typically exhibiting a sharp increase in morning hours and then a gradual decline throughout the day until bottoming out during nighttime sleep. In the present study, a participant’s treatment and control sessions either both started at 8 a.m. or 1 p.m. and this likely affected baseline cortisol levels, which may also impact cortisol reactivity to the CPT/WPT trials. To assess this possibility, a mixed-measures ANOVA was computed, with start time [AM, PM] as the between-participants factor and stress condition [treatment, control] and sample [Pre T1, Pre T3, Post T5] as within-participants factors. There were no significant effects (all *p*_null_ > 0.05; Figure 12B).

## 4 Discussion

Humans can habituate to aversive events, yet relatively little is known about the multiplex of physiological and neural responses that adaptively contribute to stress habituation. This study had two main aims. First, to simultaneously measure changes across a diverse range of variables in response to the CPT relative to the WPT. Second, to establish whether each of the variables habituates across repeated exposures, and if so, whether the timing of this habituation differs between variables. The results confirm that the CPT evoked responses across multiple physiological measures. HR, MAP, SV, CO and pupil size all increased as a function of cold-water exposure and HF, LVET and PEP decreased. Responses were also observed in the EEG oscillatory brain data across a wide range of frequency bands, with increased frontal delta and theta band activity during the CPT and increased alpha during recovery. Several cardiovascular measures also habituated across the multiple CPT exposures (HR, MAP, CO, SV and LVET) as well as pupil size and alpha-band activity across the scalp. The results confirm that multiple measures carry information about the stress response, and there is considerable variation in both the time-course of responses and whether they habituate over repeated exposures. Furthermore, robust increases in oscillatory brain activity across the theta, alpha and beta bands and dilated pupils were observed immediately prior to of cold-water exposure, thus providing several markers of anticipatory stress.

### 4.1 Cardiovascular responses

Exposure to both cold and warm water evoked an initial increase in HR, MAP and CO, reduction in SV and TPR, and shortened PEP and LVET. This response was increased as a function of cold water relative to warm water during the immersion period for several variables (HR, CO, SV, MAP, PEP and LVET). The observed increased sympathetic activity and decreased parasympathetic activity (decreasing HF throughout immersion) are consistent with an alerting/defense response that is evoked by many different forms of environmental and mental stressors (Hilton, 1982; Zbrozyna and Westwood, 1993) as well as previous investigations that use the CPT to induce stress in human participants (e.g., Lovallo 1975; Larra et al., 2015; Bachmann et al., 2018). The increase of HR, CO and MAP in the setting of reduced TPR early in the immersion period is notable. Under resting conditions, a rise in MAP, regulated through baroreceptors would evoke a fall rather than a rise in HR and a reduction in TPR. However, the baroreceptor reflex is centrally inhibited during the alerting/defense response (Mifflin et al., 1988) and is known to decrease in sensitivity with mental stress (Conway et al., 1983; Steptoe and Sawada, 1989), so this may explain why MAP and HR rise together. The initial reduction in TPR in response to cold water could be driven by vasodilation in some muscle beds and vasoconstriction elsewhere as mediated by the alerting/defense response. However, this cannot be disambiguated in the current experiment from a second thermoregulatory response to cold. Cold exposure to the skin evokes splanchnic and renal vasodilation, which could reduce global TPR (Morrison and Nakamura, 2011). The reduction of SV is also notable as it would be expected to rise with an alerting/defense response. We speculate that it is a consequence of a mixed stressor/thermal stimulus induced by CPT leading to a potential redirection of blood volume away from skin and towards the splanchnic circulation. This could reduce venous return to the heart, end diastolic volume and SV.

### 4.2 Pupillary responses

Cold-water exposure also evoked rapid pupil dilation within 10 s of exposure, followed by sustained dilation in early but not later trials. Pupil diameter changes are controlled by the dilator and sphincter muscles, which are influenced by sympathetic and parasympathetic branches of the ANS, respectively (Steinhauer et al., 2004; Bradley et al., 2008), hence these data help to further characterize the nature of the autonomic response to pain. The initial dilation is consistent with previous single-exposure data that demonstrate pupil dilation within 30 s of cold-water exposure (Tassorelli et al., 1995; Tassorelli et al., 1998). Pupil dilation was then sustained through to the end of the cold-exposure and part of the recovery period. In previous pupillometry studies the pupil returned to baseline 1–2 min into the cold-water exposure (Tassorelli et al., 1995; Tassorelli et al., 1998), whereas the present data show a sustained response in early trials. This may reflect a stronger response to immersing both feet in the present study when compared to immersing a single hand, as per the previous work (Tassorelli et al., 1995; Tassorelli et al., 1998).

### 4.3 Brain oscillatory responses

Midway through the cold-exposure timeline, changes became apparent in oscillatory brain activity. Cold-water exposure evoked an increase in frontal delta and theta activation. These patterns of activation are broadly consistent with previous work showing increased bilateral frontal delta and theta during a single CPT exposure (Ferracuti et al., 1994; Chang et al., 2002), although delta effects are noticeably more left-lateralized in the present data. These results support the notion that low-frequency EEG activity is a characteristic of the response in the human brain to the pain associated with the CPT (Knyazev, 2012), and the topography of the activation overlaps with hemodynamic imaging studies that show increased activation of pre-frontal brain regions as a function of pain (Casey, 1999; Frankenstein et al., 2001). The changes in low-frequency oscillatory activity were sustained throughout the cold-water exposure. Together, these EEG results replicate some previous findings and suggest that multiple frequency bands are involved in pain processing.

### 4.4 Habituation across cardiovascular, pupillary, brain and self-reported pain responses

Several cardiovascular variables habituated across the repeated cold-water exposures. HR was the first cardiovascular measure to habituate, in that the increase in HR was already reduced in Trial 3 relative to Trial 1, whereas the increase in MAP and CO largely habituated in later trials (i.e., Trial 5 relative to Trial 3). The habituation in both the shortening of the LVET response and the drop in SV in response to repeated cold water immersions also occurred in later trials (i.e., Trial 5 relative to Trial 3) The habituation of HR and MAP observed here is consistent with previous work in humans showing reduced HR and SBP/DBP responses to repeated exposure of the hand or foot to cold water (Glaser and Whittow, 1957; Glaser et al., 1959; Zbrożyna and Krebbel, 1985). The increase in pupil diameter also habituated early in the session (i.e., Trial 1 vs. Trial 3; note that while the habituation effect was numerically larger during treatment when compared to control, there was still significant habituation in both). Frontal-midline theta activity declined over the course of both cold- and warm-water exposures, perhaps reflecting reduced cognitive control requirements over the course of each session as participants become increasingly familiar with task demands (Cavanagh and Frank, 2014). The EEG alpha band was also modulated during repeated cold-water exposure towards the end of the exposure and recovery periods, demonstrating a complicated pattern of synchronization and desynchronization over the course of the session. Finally, subjective ratings of pain in response to cold exposure declined over the course of the study, with most of the habituation occurring between Trials 3–5. This habituation in self-reported pain is broadly consistent with previous work showing decreased pain thresholds over repeated cold exposures within a single-day session (Smith et al., 2009) and also across sessions ran on multiple days (Carman and Knight, 1992).

### 4.5 Pre-exposure pupillary and brain responses

Changes in pupil diameter and in oscillatory brain activity were observed in the period immediately prior to cold-exposure. There is evidence to suggest that pupillary activity is a physiological marker of emotional regulation (Siegle et al., 2003; Urry et al., 2006; van Reekum et al., 2007) and pupil size is also a reliable indicator of cognitive effort during the anticipation of events, with increased effort being associated with larger pupil diameter (Moresi et al., 2008) for both aversive and neutral events (Bitsios et al., 2004). The increased pupil diameter observed here prior to cold water exposure may therefore reflect a combination of anticipatory arousal and increased cognitive effort as the participant mentally prepares themselves for the forthcoming cold-induced discomfort. There was also suppression of alpha and beta activity across central EEG electrodes. This may indicate disinhibition of somatosensory cortex, which typically characterizes activated cortical regions that are preparing to process information or initiate a movement (Pfurtscheller et al., 1997), thus suggesting a link between somatosensory disinhibition and anticipation of pain. This is broadly consistent with previous work showing alpha suppression in response to observing or imagining pain in others (Whitmarsh et al., 2011; Hoenen et al., 2015), so it is possible that in the present study alpha is consistently suppressed prior to cold-water exposure trials due to participants imagining their own forthcoming pain. This effect does not habituate with repeated pain exposure.

When considering the anticipatory effects reported here, it is important to keep in mind that participants were informed at the beginning of each session which condition they would be exposed to (i.e., treatment or control) and that there would be five exposures total. Participants also had prior experience with the CPT from the initial calibration session, so in the treatment condition the knowledge that they were about to undergo five CPTs likely increased their anticipatory stress levels throughout the entire session. Furthermore, the repeated exposures to the CPT may have compounded anticipatory stress effects throughout the session.

Changes across several cardiovascular measures were also observed in the time-period prior to both cold and warm water exposures. While it is possible that changes in these pre-exposure measures may in part be driven by anticipation of the forthcoming exposures, any anticipatory effects from the second trial onwards are likely conflated with carry-over responses from previous trials, so we cannot make any strong statements about anticipatory stress effects *per se*.

### 4.6 Cortisol is not sensitive to repeated cold-water exposure

Elevated cortisol at the outset of both treatment and control conditions and prior to any CPT/WPT exposures indicated that anticipation of both sessions induced stress in participants. Prior experience with the CPT in an earlier calibration session, as well as the ∼ 1 h of instrumentation at the beginning of the session may have contributed to the stress. The lack of interaction between treatment and control in later samples indicates that cortisol was not sensitive to repeated CPT exposures, which is broadly consistent with previous work demonstrating no cortisol response to a single unilateral arm CPT exposure (McRae et al., 2006; Duncko et al., 2007; Schwabe et al., 2008) and suggests that repeated CPT exposures do not activate the HPA. However, it has been previously demonstrated that a single 3-minute CPT (both feet) can evoke a robust cortisol response (Bachmann et al., 2018), so it is possible that the 90 s CPT exposures used in the present study were not sufficient to evoke a strong HPA response. Furthermore, it is possible that elevated stress at the beginning of both sessions may have masked any CPT evoked responses. Experimental designs intending to use cortisol as an index of HPA activation by the CPT should be mindful of lab-visit induced stress effects as well as CPT duration.

### 4.7 Limitations and future directions

It is important to acknowledge that the present dataset was collected as part of a larger study into the effects of stress on the human brain and behavior, so the CPT protocol and suite of measures were optimized for the main goals of that study i.e., investigating how CPT exposure impacts subsequent performance across a battery of cognitive tasks and the physiological correlates of any adaptive behavioral changes. There are several limitations associated with this dataset and our findings that must therefore be discussed.

First, the cognitive tasks completed by participants between CPT/WPT exposures as part of the larger protocol may have confounding effects on the stress responses and this may have impacted results presented here. Furthermore, the duration of the cognitive tasks varied, meaning that the recovery time between CPT/WPT exposures was not entirely consistent throughout each session, which may have also impacted the results. Second, participants were also not asked to modify their consumption of caffeinated beverages prior to testing sessions, as the main testing sessions were long and it was a concern that requiring them to abstain from caffeine may lead to excessive sleepiness with detrimental effects on cognitive task performance. However, it is important to acknowledge that differences in caffeine levels between individuals may influence the cardiovascular responses to the CPT. Third, no attempt was made to test female participants at a specific stage of the menstrual cycle and that variability in estrogen and other hormone levels may impact our results. Fourth, given the scale of the larger BOSS study and the length of each testing session, it was necessary to test each participant in either morning sessions starting at 8 a.m. *or* afternoon sessions starting at 1p.m. Many cardiovascular variables such as HR, HF, BP and CO are known to have circadian rhythms (Millar-Craig et al., 1978; Furlan et al., 1990; Yamasaki et al., 1996; Guo and Stein, 2003) so it is possible that the variability in baseline values may have affected both anticipatory and evoked responses to the CPT manipulation. Fifth, the repeated measures design led to systematic baseline trends in several cardiovascular variables and the precise source of these trends is difficult to determine. Our main analyses were baseline corrected, but it is still possible that these trends may have impacted the outcome of our results. Despite these limitations, the unique combination of measures and multiple CPT exposures within a session provide a novel and valuable opportunity to study stress habituation across multiple physiological and neural responses.

We also acknowledge that there is growing interest in establishing which cortical regions have the most influence over ANS, and anatomically there is evidence from human fMRI and intercranial EEG studies that regions such as insular and anterior cingulate cortex are major contributors to both sympathetic and parasympathetic responses (e.g. Macey et al., 2012; Cechetto 2014; Pal et al., 2021; Roquet and Conti 2021). Here, we do not attempt to explore any direct relationship between our scalp recorded EEG measures and ANS modifications as this is a complex question that is beyond the scope of this paper, but we are interested in exploring this relationship in future analyses of our data.

### 4.8 Summary

The goal of the present study was to interrogate the multiplex of physiological and neural responses to repeated cold- or warm-pressor tests using an extensive suite of measures. The findings elucidate the time-course of stress effects and habituation over repeated exposures in multiple cardiovascular, pupillometry and EEG measures, providing valuable insight into anticipatory and evoked stress effects on the ANS and CNS.

## Data Availability

The raw data supporting the conclusions of this article will be made available by the authors, without undue reservation.
