# Supplementary material for: Habituation of the stress response multiplex to repeated cold pressor exposure
Source: Front Physiol. 2023 Jan 10;13:752900. doi: 10.3389/fphys.2022.752900 (PMC9871365; doi:10.3389/fphys.2022.752900)
Supplement: Supplementary file 1 [file Image1.pdf]

Habituation of the Stress Response Multiplex to Repeated Cold Pressor Exposure

Supplemental Information

Fig S1. Within trial analyses for cardiovascular variables. To determine whether a change occurred *within* either condition for all cardiovascular variables we computed additional ANOVA analyses that incorporated change in baseline as a factor, comparing data recorded during the pre-CPT baselines with data recorded during and after the CPT exposures. The raw data for each measure are plotted in panels below. We computed 2 [baseline: pre-CPT, during/post CPT] x 2[condition: treatment, control] x 5 [trial: T1>T5] ANOVAs for all timepoints ranging from the start of the immersion period (65 s) onwards. Given that in each test the baseline data were averaged over 15s, for consistency a 15 s moving average window was also applied to the data from each immersion and recovery timepoint (i.e. 65-155 s), hence the statistical comparisions terminate at 180 s. Consistent with our main cardiovascular analyses, the complete results from the ANOVAs are plotted above in the panels above. Each panel contains a separate plot for the treatment and control condition, with the mean response across participants in each repetition trial (T1-5) represented in each plot by a different color (ROYGB). Horizontal lines positioned between treatment and control plots indicate statistically significant ANOVA main and interaction effects for the baseline, condition and trial factors. Note that the figure is split across two pages to facilitate viewability.

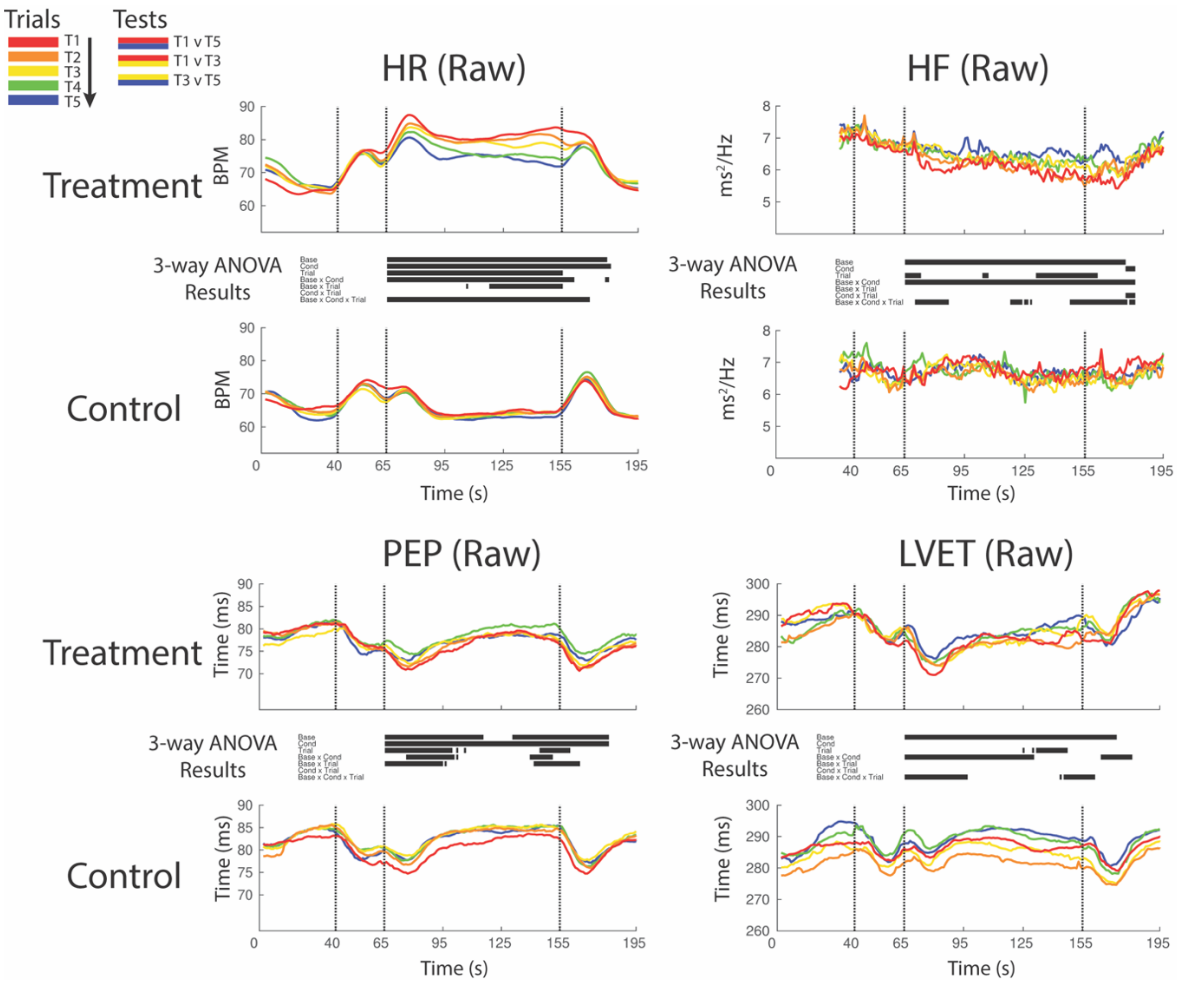

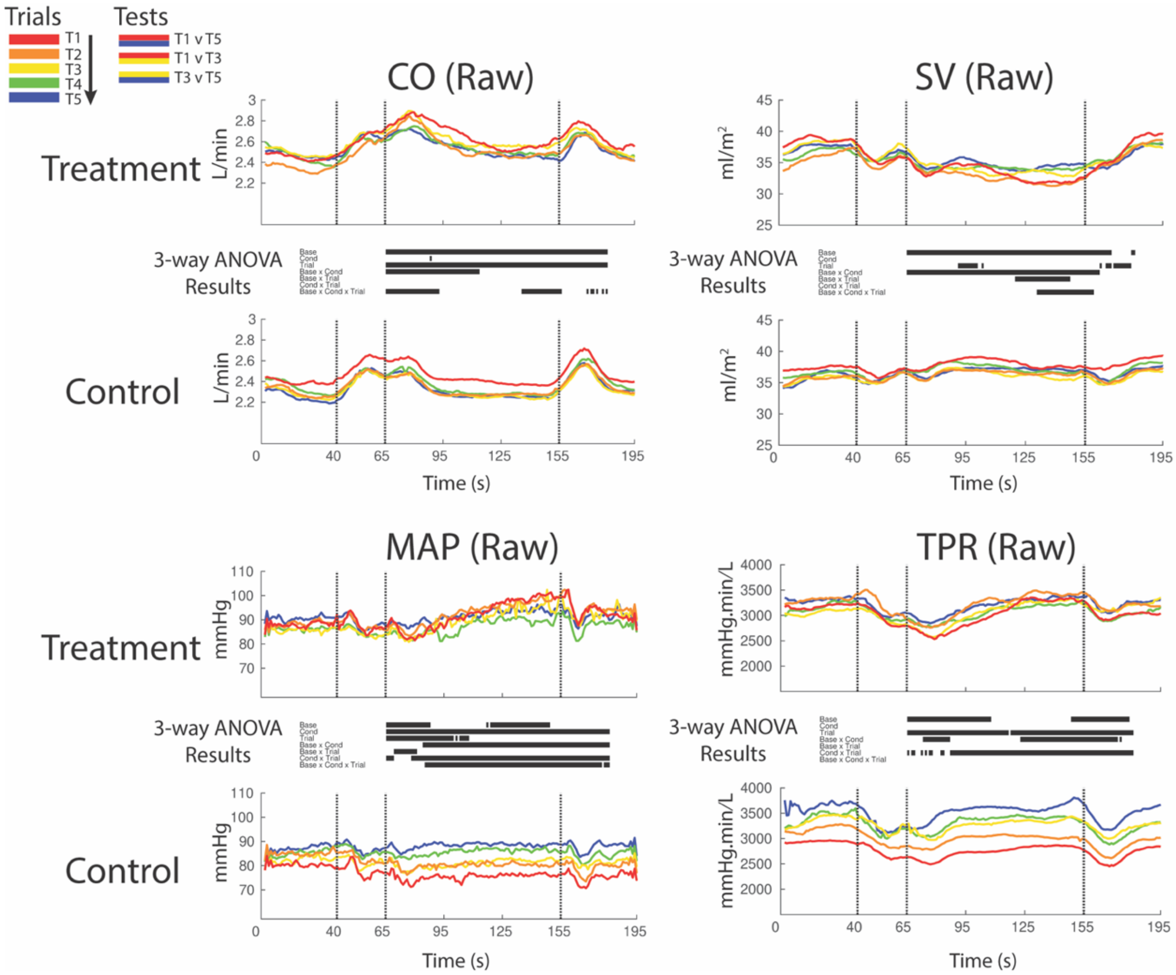

Trials
T1
T2
T3
T4
T5
Tests
T1 v T5
T1 v T3
T3 v T5
CO (Raw)
SV (Raw)
MAP (Raw)
TPR (Raw)
Treatment
Control
3-way ANOVA Results
Base
Cond
Trial
Base x Cond
Base x Trial
Cond x Trial
Base x Cond x Trial
L/min
ml/m²
mmHg
mmHg.min/L
Time (s)
